# Volcanic processes within the Petavius crater, nearside of the Moon

**DOI:** 10.1038/s41598-025-95132-5

**Published:** 2025-03-24

**Authors:** A. V. Satyakumar, Shreekumari Patel, Deep Dixit Patel

**Affiliations:** 1 CSIR-National Geophysical Research Institute (CSIR-NGRI), Hyderabad, 500007 India; 2 Academy of Scientific and Innovative Research, Ghaziabad 201002, India; 3 Space and Planetary Science Group, Department of Earth Sciences, Khalifa University, Abu Dhabi, United Arab Emirates; 4 Department of Earth Sciences, University of Western Ontario, London, Canada

**Keywords:** Volcanism, Bouguer anomaly, GRAIL, Moon, Mafic minerals, Petavius, Planetary science, Geochemistry, Mineralogy

## Abstract

This study presents a detailed investigation of compositional data (e.g., Moon Mineralogy Mapper (M^3^), Kaguya mineral abundance map) and gravity data (Regional, residual, and crustal thickness) to understand the volcanic history of Petavius crater (~ 180 km diameter crater centered at 25.3° S, 60.4° E). Spectral analysis of M^3^ data reveals the presence of feldspar mineral-plagioclase and olivine at the central peak and other mafic minerals like high and low pyroxene in distinct mare units. The composition of volcanic units are mafic with dominant phases of olivine, pyroxene and plagioclase mineral. Primary melt compositions simulated using the COMAGMAT model suggest that the magmas are Mg-rich (MgO = 10.3–24.5 wt%), and the thermal regime of the Petavius mantle was not anomalously hot (i.e., > 1500 °C). Bouguer gravity anomalies show an irregular high (~ 20 mGal) over the central peak towards the southwest of the crater and a minor poorly spread associated negative annulus. The fractures on the floor are probably linked with an underlying magmatic sill of high density, which may be affecting the observed positive Bouguer Gravity Anomaly patterns. The crustal thickness varies from 27 to 29 km at the center, 30–32 km at the floor, and 33–39 km at the rim of the crater. Considering all these points, we conclude that mare material that erupted through floor fractures of the crater floor was likely the result of magmatic intrusion within the fractured crust underneath the crater floor.

**Supplementary Information:**

The online version contains supplementary material available at 10.1038/s41598-025-95132-5.

## Introduction

The most notable geological process that has changed the entire surface of the Moon is impact cratering. Following the primordial impact events, volcanic activity stands as the next predominant geological force that has significantly transformed the lunar surface (e.g.,^1,2^). Due to numerous geodynamic processes and conditions, magma is carried from the interior of the Moon to the surface during volcanism^3–5^. When the denser rock pools on the surface, it induces local stress fields in the underlying bedrock that cause preexisting faults to reactivate and arcuate rills to form^6,7^. These forces also create mare ridges and floor-fractured craters (FFCs). The best examples of magma transportation, emplacement of lava, and tectonic activity due to volcanism on the Moon are the FFCs. As inferred from^8^ Michaut et al. (2020), dense mare basins and mascon nearby significantly contributed to the lithospheric stress field, enabling an upward magma ascent to the shallow depth of the crust. Below a volcanic structure, the activated compressive stress resists the rise of magma, only permitting the upsurge of the least dense and, consequently, most differentiated magma^9^. FFCs are the perfect example of the magmatic, thermal, and morphological evolution that has shaped the present Moon^3,10,11^. Furthermore, they offer exceptional extraterrestrial volcanic environments to test how the various mechanisms suggested to affect magma transit and shallow emplacement on the Moon also hold across the solar system^12^.

Most FFCs are found close to the large basins due to basin formation processes, such as extensive fracturing and deformation from basin-scale impacts, which create pathways for magma intrusion. Craters such as Gassendi (~ 110 km diameter, Class 3) and Petavius (~ 180 km diameter, Class 1), which are situated in highland regions adjacent to Mare Humorum and Mare Fecunditatis respectively, exhibit fractured floors^3^. Extensive research on Gassendi Crater has unveiled significant insights into its geological features, including lava lakes and mare emplacement attributable to magma intrusion and emplacement on the surface, multiple volcanic event, mineralogical diversity (presence of pyroxene, olivine and plagioclase), distinct morphological characteristics (rilles, central peak and mare deposits), etc.^13–15^. The scientific literature presents a limited number of investigation studies that focus on the Petavius crater in conjunction with other craters characterized by floor fracturing^10,16–22^. On the Moon, pyroclastic deposits, small lava flows, and evidence of magmatism are often located within impact craters^11,23^. A surface unloading caused by an impact crater provides a driving overpressure to the magma stalling at depth. This overpressure counterbalances the melt negative buoyancy, favoring its ascent through the crust. Magma ascends up to the crater floor for a large unloading on a thin crust or alternatively forms an intrusion. Therefore, small impact cratering likely induced magmatism and thereby crustal evolution in the early times of terrestrial planets^24^. Eruption at the surface was possible if magma was stalling below a relatively thin crust and a large crater and was still connected to a deeper source or forming an independent vertically elongated storage zone^24^. For smaller craters and/or thicker crust, the overpressure was smaller and magma formed an intrusion^24^. Therefore, we selected the Petavius crater to investigate in detail to understand the compositional diversity within the crater, volcanism, and origin source for the rilles and flows using remote sensing and gravity datasets.

**Fig. 1:**
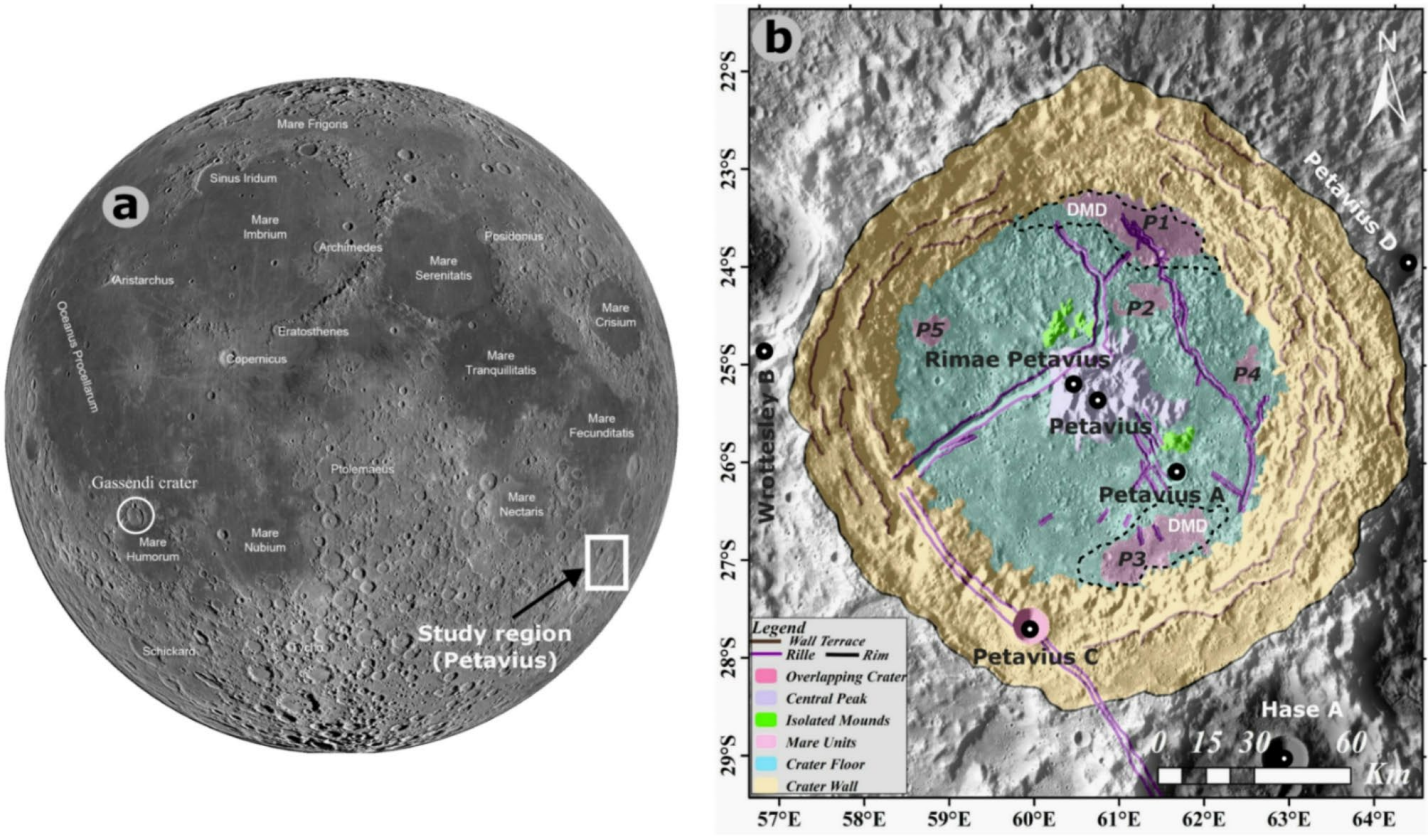
(**a**) WAC mosaic of the Moon nearside with major basins/crtaers marked. The white rectangle is the study area (Petavius crater) and white circle is the Gassendi crater (**b**) Mapped geomorphological features of the Petavius crater showing Rille, Central peak, isolated mounds, Mare units, Crater floor, Crater wall, and Scraps at wall terrace (Projection: Stereographic centered at the Petavius crater). Figure is prepared using the global WAC image of the Moon using ESRI ArcGIS 10.4. The drawing, like rille, rim, wall terrace, central peak, mare units, isolated mounds, crater floor, and crater wall are mapped using the ESRI ArcGIS 10.4 by applying various image stretching techniques, including Gamma stretch, Maximum stretch, and Minimum stretch to get a good contrast image. The DMD (dark mantling deposits) boundaries (dotted polygons) are adopted from Gaddis et al. (2013) and Gustafson et al. (2020).

Petavius crater (25.3° S, 60.4° E), a ~ 180 km diameter crater, is one such floor-fractured crater situated SE of Mare Fecunditatis adjacent to the southeastern limb of the Moon (Fig. 1a,b), an altered type of crater as a result of later volcanism, uplift, and fracturing. This crater is one of the most fascinating on the Moon, modified by post-impact processes. The mare occupies the central floor, and numerous dark mantling deposits were found all over the floor (e.g.,^19^Gustafson et al., 2020), suggesting volcanism may have influenced the crater’s appearance, but to what extent? After the crater was created, the floor’s molten rock began to rise upward, breaking the crust and only releasing small floods of basalt that remain stain the floor today. Rimae Petavius and the dark mantling deposits from the post-impact volcanism were still significant. What could have caused the Rimae Petavius fracture system, which sliced the floor? Why did the fracture system in the Petavius crater become so extensive? Why Petavius crater is not flooded with basalt is the more challenging question. The eruption at Petavius crater likely differed from those in other parts of the Moon. For some reason, the magma beneath the Petavius crater might not have been buoyant enough to flood the surface entirely. Finally, it is possible that only a limited amount of basalt was erupted because the magma source region was so small. As a result, these observations and the earlier research have enabled us to thoroughly examine the numerous as yet unrevealed aspects of Petavius and lunar volcanism. This paper aims to investigate the compositional variations and spectral signatures of newly identified mare units within the crater and understand the impact induced magmatism.

## Results

### Compositional variations

**Fig. 2:**
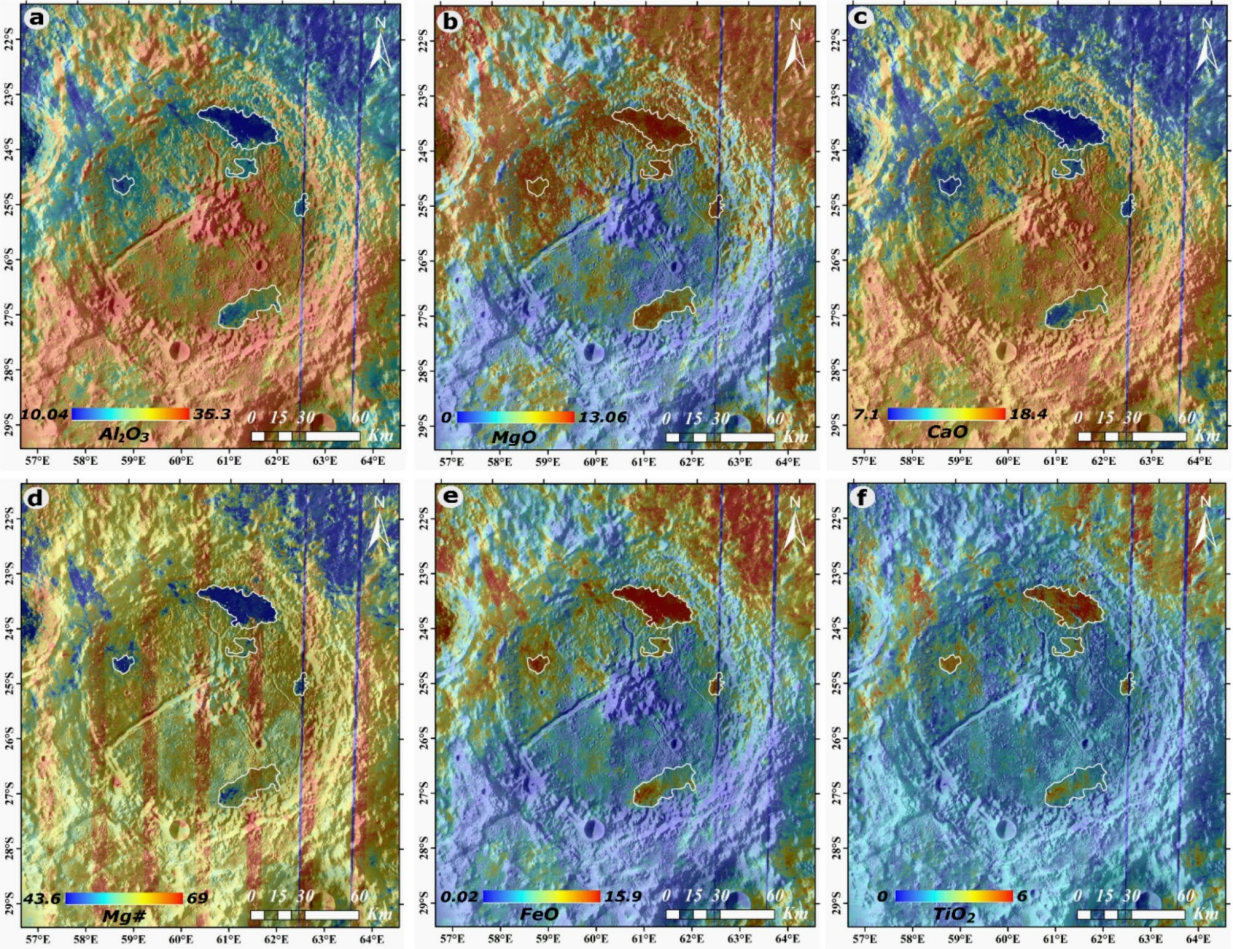
Abundance maps (**a**) Al_2_O_3_, (**b**) MgO, (**c**) CaO, (**d**) Mg#, (**e**) FeO, (**f**) TiO_2_ of the Petavius crater^25,26^. The basemap for panel (b)-(h) is WAC global hillshade (Projection: Stereographic centered at the Petavius crater). The global lunar surface major oxides and Mg # abundances are downloaded from Zhang et al. (2023) and Ma et al. (2022) and extracted for the region of interest in this study (Figure) using Generic Mapping Tools (GMT) software (Wessel et al., 2013). After extraction, we used ESRI ArcGIS 10.4 to prepare Figure. The DMD (dark mantling deposits) boundaries (polygons) are adopted from Gaddis et al. (2013) and Gustafson et al. (2020).

**Fig. 3:**
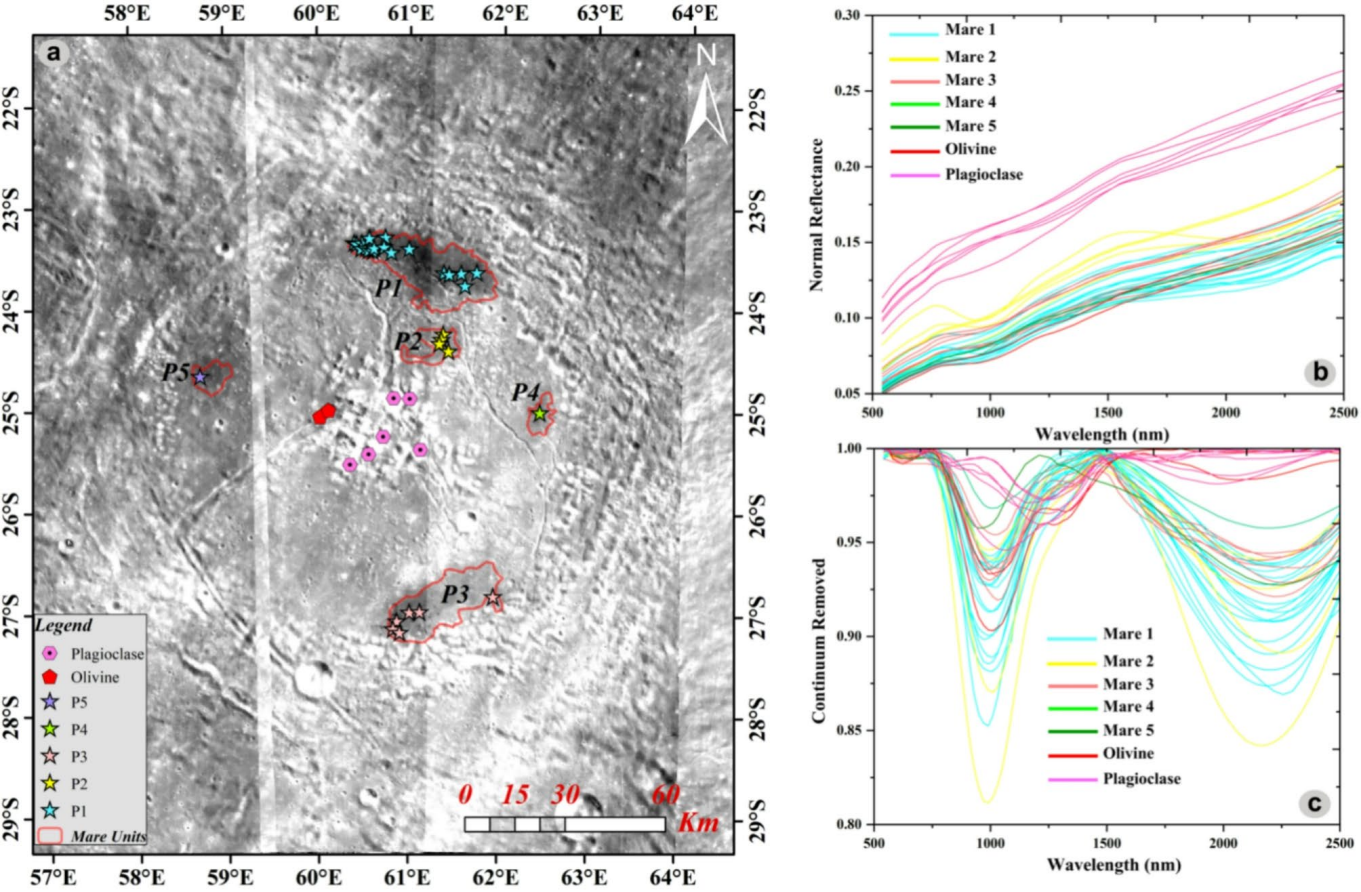
(**a**) The M^3^ R1.573 Albedo map exhibits a comprehensive representation of various mare units. The image incorporates stars, pentagons, and octagons to symbolize the locations where spectra were obtained. The solid red outlines demarcate the distinct boundaries of the individual mare units (Projection: Stereographic centered at the Petavius crater), (**b**) Normal Reflective signature of samples, (**c**) Continuum Removed signatures.

The chemical composition of the lunar surface under investigation, as ascertained from the abundance maps of elemental oxides^25,26^, reveals distinct characteristics for the basaltic units within the crater (Fig. 2a–f). The spatial boundaries of these basaltic units are delineated based on variations in elemental oxide concentrations relative to the surrounding terrain. The defined dark mantle deposits are referenced from previous investigations of the Petavius crater by Gustafson et al. (2020). Specifically, these units exhibit 15.4–20.6% Al_2_O_3_, 8.9–10.8% MgO, 11–13% CaO levels, and 9.2–15% FeO content while showcasing a 0–4% of TiO_2_. A comprehensive breakdown of the precise oxide percentages concerning the individual basaltic units can be found in Table 1. P1-P5 values in Table 1 correspond to locations shown in Fig. 1b. The non-basaltic region prominently showcases an elevated concentration of Al_2_O_3_, MgO, and CaO (Fig. 2a–c), indicating a pronounced prevalence of these oxides in this particular terrain (i.e., central peak, crater wall, and ejecta). The dark mantle deposits to the north, which also enclose the mare unit P1, exhibit a significantly elevated mafic composition, with FeO concentrations reaching up to 15 wt% and TiO_2_ levels as high as 4 wt%. The highest concentrations in P1 occur near the rille complex at the center of the deposits and around impact craters in the area, likely exposing mafic material from beneath a surface layer covered by highland ejecta. In contrast, the dark mantle deposits to the south, surrounding the P3 mare unit, show a more moderate mafic composition, with FeO concentrations up to 11 wt% and TiO_2_ reaching 2.2 wt%, primarily associated with dark-halo impact craters (craters markes as stars in Fig. 3). These values align with previous findings by Gustafson et al. (2020). Further, along the southwest-trending rille from the central peak complex, distinct iron-enriched anomalies and an olivine signature (as identified in M^3^ spectral data) are observed. These mafic deposits suggest a history of volcanic activity, consistent with the interpretations of Gustafson et al. (2020).

**Table 1:** The elemental oxide abundance values of basaltic units of the Petavius crater extracted from Ma et al. (2022) and Zhang et al. (2023).

Mare units	Al_2_O_3_ (wt.%)	MgO (wt.%)	CaO (wt.%)	FeO (wt.%)	TiO_2_ (Wt.%)	SiO_2_ (wt.%)
P1	15.6–20.4	8.9–10.78	11.4–13.0	9.2–15.0	0.0–4.0	40.1–48.27
P2	15.4–20.6	9.0–10.3	11.0–12.8	8.2–10.8	0.16–1.45	43.64–45.24
P3	17.3–19.4	9.2–10.4	11.8–12.6	9.6–11.0	0.05–2.12	40.8–45.2
P4	19.83–20.0	9.67–10.0	12.48–13.0	10.16–10.5	1.83–2.0	45.76–46.0
P5	19.73–20.0	8.97–9.0	12.72–13.0	9.65–10.0	1.99–2.0	40.77–41.0

Conversely, as depicted, the basaltic units exhibit relatively higher percentages of FeO and TiO_2_ (Fig. 2e,f), setting them apart from the surrounding morphological features of the crater. The abovementioned data leads to the compelling inference that P1-P5 units are predominantly composed of 0.1–4% TiO_2_ and 15.4–20.4% Al_2_O_3_ basalt, bearing a striking resemblance to the geological composition found in the nearby Mare Fecunditatis Region^27,28^. This intriguing correlation between the basaltic formations strongly suggests a noteworthy geological affinity, implying potentially shared origins or geological evolution between these areas.

### Mineralogical features from M^3^ Spectra

Spectral investigation of M^3^ data reveals the distribution of lunar primary minerals like plagioclase, olivine, and pyroxene across the different morphological features of the crater. We detected a concentration of high albedo minerals at the central peak of the crater (Fig. 3a). The pink hexagons in Fig. 3a delineate precise locations from which spectra signatures were meticulously collected within the region of interest. These reflective signatures have single board minima at ~ 1.25 µm exposing the feldspathic mineral-plagioclase (Fig. 3b), indicative of upper anorthositic crustal rock. The red pentagons represent an aggregation of mafic minerals with a broad minimum of ~ 1.05 µm and no or weak spectra minimum around ~ 2 µm. The spectral characteristic is compatible with the mafic mineral-olivine signature (visually represented in Fig. 3a–c). The significance of the spatial distribution of the red pentagons lies in their positioning along the rille of the crater, indicating the likely existence of an olivine-rich source, possibly olivine-basalt. The star polygons in Fig. 3a indicate specific M^3^ low albedo sites strategically chosen for collecting pyroxene spectra. These spectra are recognized by their characteristic absorption features occurring around ~ 1 µm and ~ 2 µm, signifying the occurrence of pyroxene minerals at those precise locations.

**Fig. 4:**
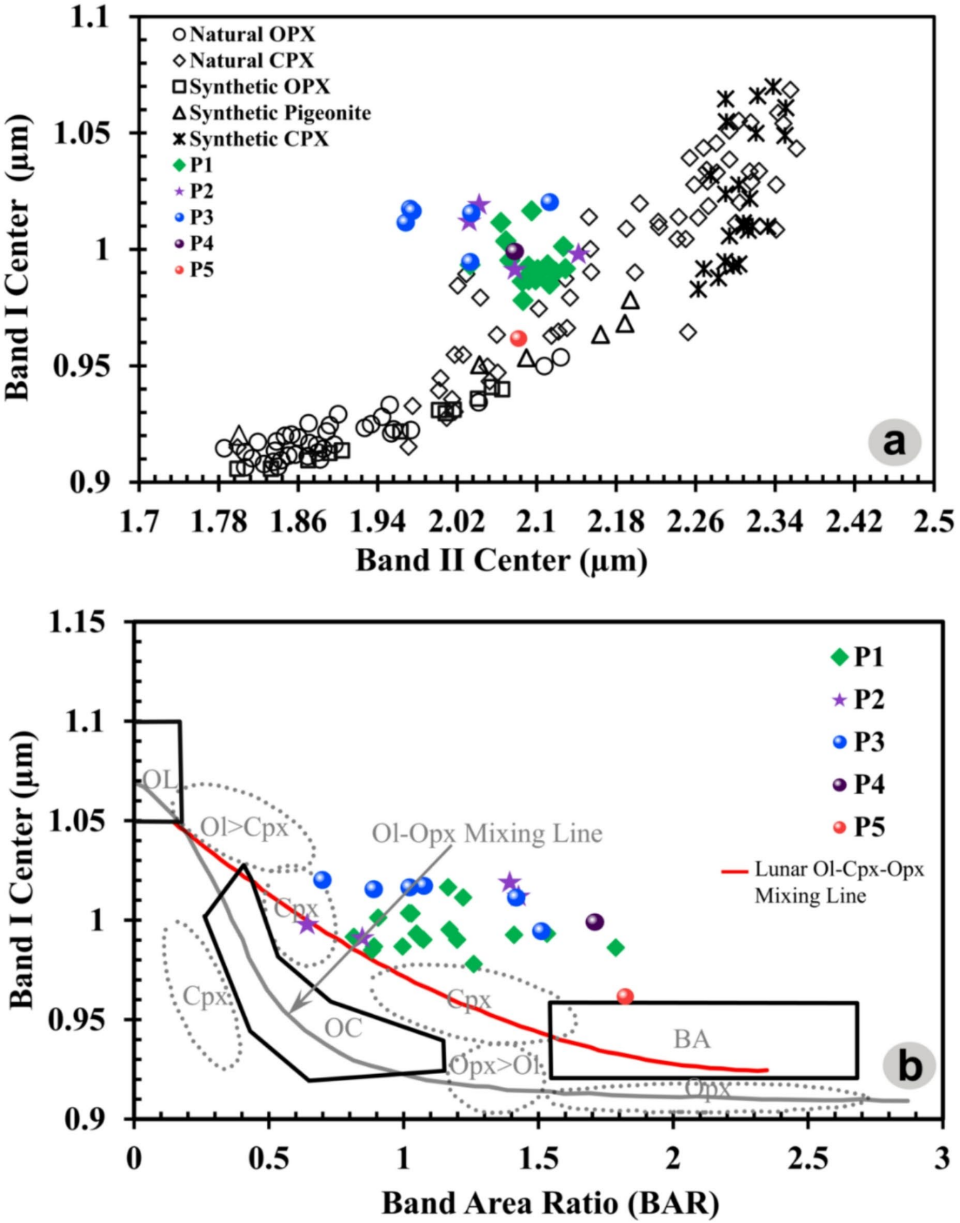
(**a**) The BC-II vs BC-I (corrected) graph represents pyroxene spectra originating from different mare units, and (**b**) The graph illustrating BAR vs BC-I, as per the approach detailed by Gaffey et al. (1993). The OC polygon encompasses the mafic silicate composition of ordinary chondrites, while the BA rectangular region denotes pyroxene-dominated basaltic achondrites (Gaffey et al., 1993). The OL-Opx line delineates the range of band minima for pure olivine and orthopyroxene as a function of their iron content^31^. The red Lunar-OL-CPX-OPX mixing line aids in classifying types of lunar rocks; data points above this line indicate Ilmenite and mafic-rich rocks, whereas points below suggest compositions rich in glass and plagioclase^32^.

To differentiate the specific type of pyroxene present within the mare units (P1-P5 in Figs. 1b and 3a), an analysis involved plotting the band parameters on two comprehensive graphs: BC I vs. BC II (Band Center I vs. II) and BAR vs. BC I (Band Center I) (Fig. 4a,b) is carried out. These graphical representations yielded valuable insights, enabling a characterization of the pyroxene types, clearly distinguishing between Low-Ca Pyroxene (LCP) and High-Ca Pyroxene (HCP). In the characterization of these units, the P1 unit is distinguished by its prominent BC-I data spanning the spectral feature of ~ 0.98 µm to ~ 1 µm, complemented by distinctive BC-II data observed across the range of ~ 2.03–2.12 µm (Fig. 4a). Similarly, the mare unit P2 is marked by discernible BC-I data encompassing ~ 0.99 µm to ~ 1.01 µm while displaying distinct BC-II data within the spectral range of ~ 2.03–2.14 µm (Fig. 4a). The P3 unit, on the other hand, reveals significant BC-I data observed between ~ 0.99 µm and ~ 1.02 µm, alongside characteristic BC-II data extending from ~ 1.97 to ~ 2.11 µm. The P4 unit showcases an intriguing spectral feature in contrast to the previous units: BC-I and BC-II manifest at a wavelengths, specifically around ~ 0.99 µm and ~ 2.08 µm, respectively (Fig. 4a). Lastly, the P5 unit exhibits a noteworthy variation, with its BC-I data evident at ~ 0.96 µm, while its distinctive BC-II is observed at ~ 2.08 µm (Fig. 4a). Figure 4b showcases a meticulous plot, incorporating the pyroxene values sourced from^29,30^ and the examined pyroxene data. The plot depicts a discernible compositional trend, shifting from Low-Ca to High-Ca pyroxenes. The results elucidate that units P4 and P5 display notable compositional resemblances characteristic of LCP, whereas the remaining units exhibit chemical affinity with HCP (sub-calcic to calcic-augite mineral).

For a comprehensive understanding of the rock composition characterizing the coexistence of olivine and pyroxene minerals within the acquired reflectance samples, the data was plotted on the BAR vs. BC-I graph (Fig. 4b). The data analysis revealed that the mare composition primarily corresponds to the pyroxenes, which dominate the region of interest. A notable observation pertains to the five basaltic units within the Petavius crater. In these reflectance spectra, specific datasets demonstrated BAR values ranging from ~ 0.64 to ~ 0.9, indicative of mineralogy dominated by olivine. In addition to the previously mentioned data, it was observed that the BAR values for other data varied between approximately ~ 0.9 to 1.5, with a few points even surpassing the 1.5 threshold. This pattern strongly indicates a substantial presence of High-Ca Pyroxene and Low-Ca Pyroxene minerals in these units. Moreover, a discernible trend was observed, wherein the BAR value showed an incremental rise in values with the increasing dominance of pyroxene minerals in the spectral data. This insightful observation indicates a progressive shift from olivine to pyroxene dominance, elucidating the dynamic mineralogical transformations within the studied samples.

### Composition of mafic units and melts

**Fig. 5:**
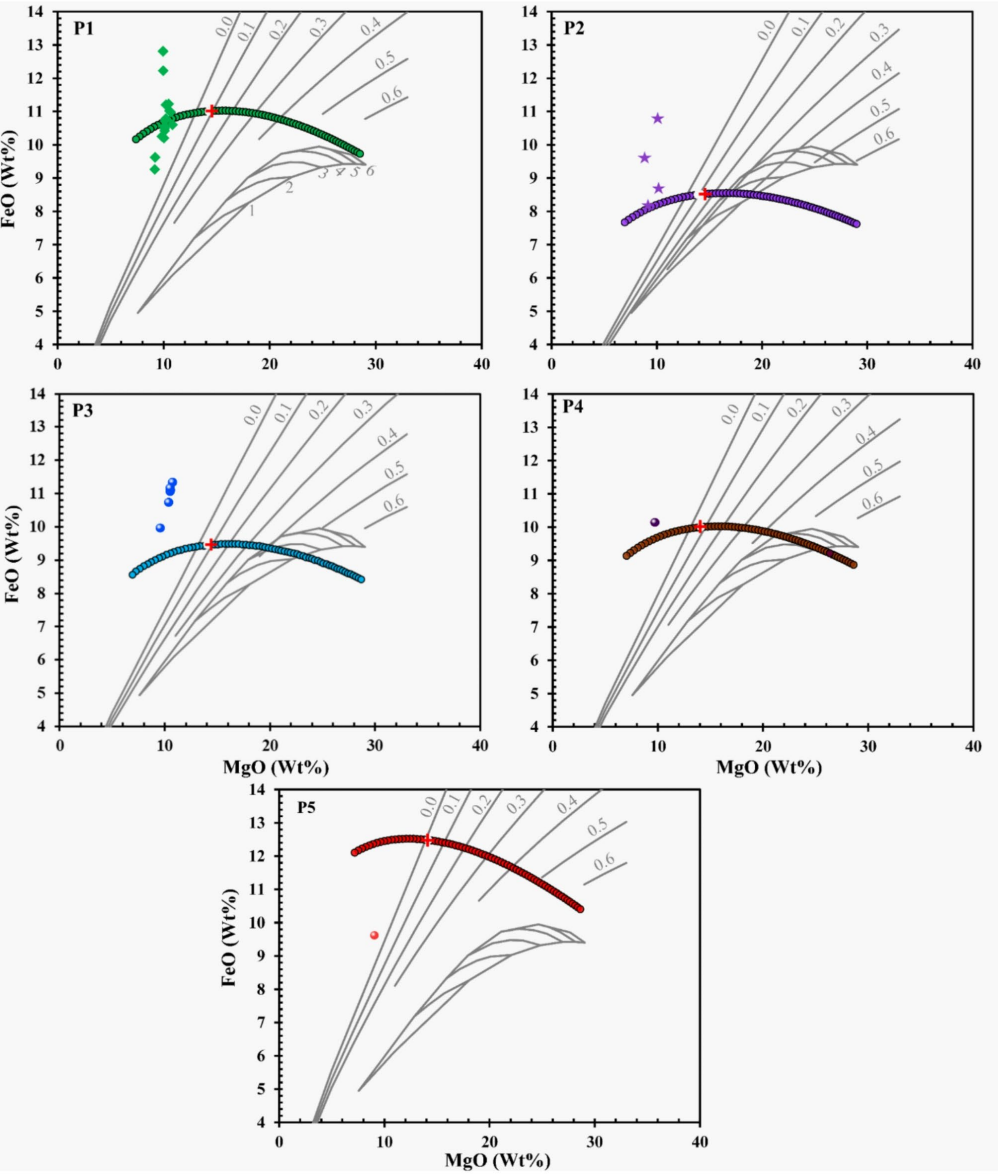
FeO–MgO relationships in the context of mantle melting deposits. The diagram illustrates the comprehensive spectrum of attainable liquid compositions achievable through the utilization of olivine and harzburgite as residual assemblages. The harzburgite cotectic depicted in the figure at 1–6 GPa serve to delimit the pressure range for the anticipated liquid compositions.

This section encompasses the inverse modeling aspect, which defines the range of potential primary magma compositions depicted in Fig. 5. In parallel, the forward modeling component, presented in Fig. 5, exemplifies the concept of accumulated perfect fractional melting from a presumed magma source. The binary projection involving FeO and MgO proves instrumental in determining the primary magma composition, concurrently yielding insights into the associated melt fraction(F). In the FeO vs MgO plot, the position of data points can provide information about its mineral composition. The points what shift along the FeO-MgO line could indicate a change in the relative proportions of these two oxides during crystallization. The plus sign indicate the melt fraction value in the plot. A shift towards higher MgO and FeO contents could indicate a higher formation temperature. A higher melt fraction could potentially lead to a shift towards higher FeO contents, as iron is relatively incompatible element.Liquids resulting from equilibrium melting exhibit deficient FeO levels in comparison to the primary magma. Enhanced congruence is observed in instances of accumulated fractional melting where olivine serves as the sole residuum phase. In unit P1, a melt fraction 0.102 yields a primary magma with 14.23% MgO and 11.02% FeO, with olivine composition Fo87.7 and Mg# 0.67. In unit P2, a melt fraction 0.127 results in 14.234% MgO and 8.51% FeO, with olivine composition Fo89.8 and Mg# 0.71. Unit P3 has a melt fraction of 0.083, resulting in 14.03% MgO and 9.46% FeO, with olivine composition Fo89 and Mg# 0.69. For unit P4, a melt fraction of 0.077 gives 13.902% MgO and 8.9% FeO, with olivine composition Fo88.6 and Mg# 0.68. In unit P5, a melt fraction 0.021 yields 14.15% MgO and 12.48% FeO, with olivine composition Fo88.4 and Mg# 0.64. The Fe and Mg# values for olivine mineral are calculated by COMAGMAT application^33^.

**Fig. 6:**
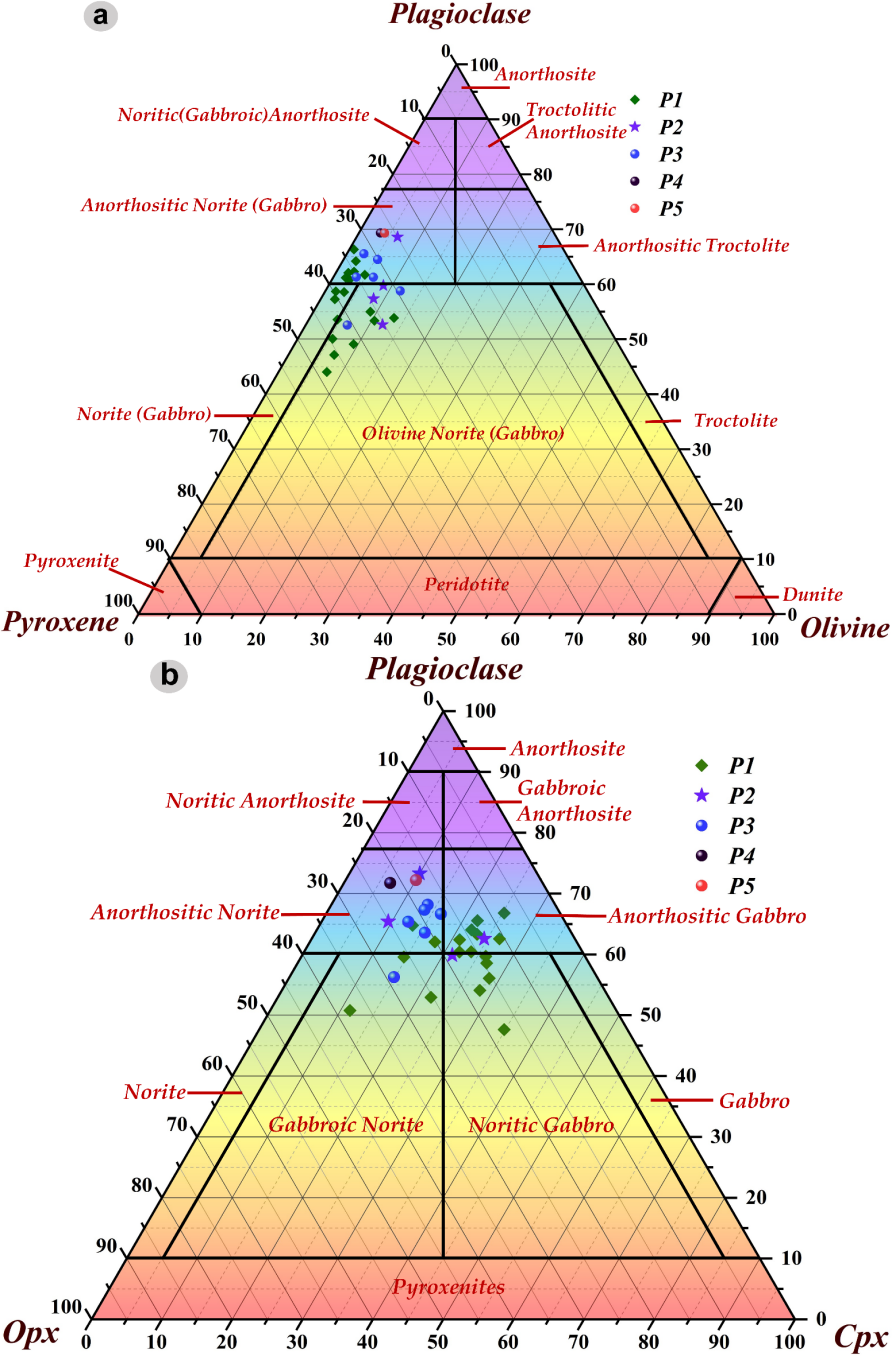
Stoffler Ternary diagram (**a**) Plagioclase-Pyroxene-Olivine, and (**b**) Plagioclase-Opx-Cpx diagram enabling the systematic classification of mafic units (Mare units) located within the Petavius crater. Within this framework, abbreviations Opx and Cpx, respectively, represent Orthopyroxene and Clinopyroxene in this context.

Herzberg and O’Hara (2002)^34^ noted a significant disparity in melt fractions, ranging from 0.11 for a depleted source to 0.28 for a fertile source. Notably, all primary magmas consistently position themselves within the stability field of Melt + Olivine + Opx, closely aligning with the melt fraction linked to Cpx-out in both projection and FeO-MgO plots^34^. Primary magmas across all units are constrained within the range of 13.9% to 14.23% MgO. Melt fractions exhibit a spectrum from 0.021 to 0.127, while the calculated olivine compositions yield mg-numbers from 88.4 to 89.8. The magmatic systems with a high melt fraction (> 0.5) exhibit a more fluidic nature. This implies that these systems possess a lower viscosity, and a greater proportion of the magma is molten, thereby increasing the likelihood of an eruption. Conversely, magmatic systems with a lower melt fraction are characterized by a higher viscosity and a greater proportion of solid crystals relative to the fluid content^35^. These observations strongly suggest the occurrence of porphyritic rock in these specific mare units. The percentage compositions of plagioclase, olivine, clinopyroxene, and orthopyroxene within the Petavius basaltic units were extracted from the Kaguya mineral abundance map, as detailed in the work by Lemelin et al. (2019)^36^. These values were plotted on Stoffer’s ternary diagram, a recognized classification method for mafic rock, as detailed by Le Maitre et al. (2002)^37^. Upon analyzing Fig. 6a, it becomes apparent that the compositional trend of P1, P2, and P3 units progresses from olivine-gabbro to anorthositic-gabbro, whereas P4 and P5 units exhibit similarity to anorthositic-gabbro. Continuing with Fig. 6b, P1 and P2 units predominantly represent noritic gabbro to anorthositic gabbro, while P3 demonstrates a trend from gabbroic norite to anorthositic norite. Conversely, P4 and P5 units align with a compositional trend associated with anorthositic norite. These findings provide valuable insights into the diverse mineralogical attributes and distinct geological evolution characterizing the basaltic units within the Petavius crater.

### GRAIL gravity signatures, isostatic regional, and residual anomalies

**Fig. 7:**
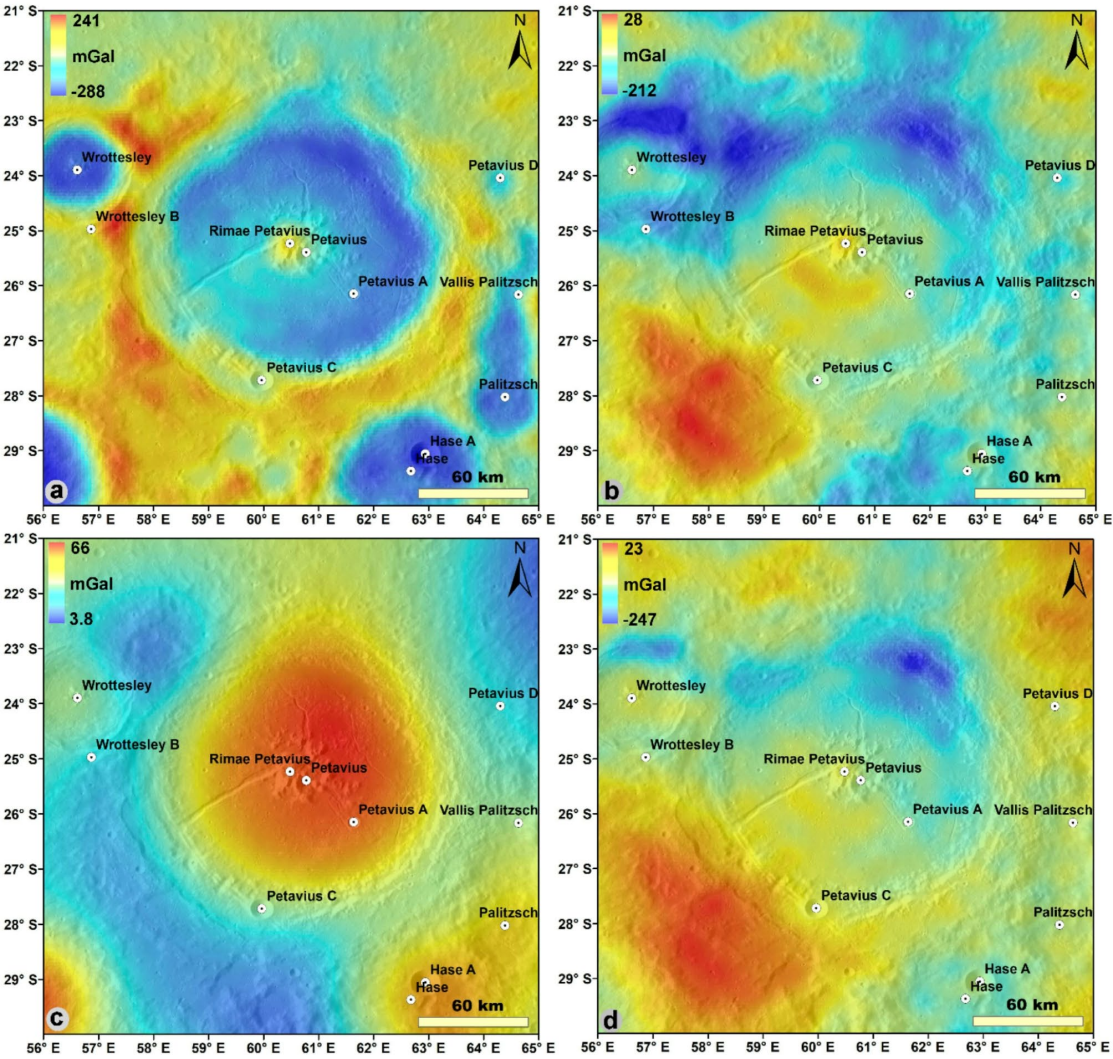
(**a**) Free air gravity anomaly map of the, (**b**) Bouguer gravity anomaly map of density 2550 kg/m^3^, (**c**) Regional gravity anomalies of the Petavius crater, and (**d**) Residual gravity anomalies of the Petavius crater. The gravity model (JGGRX_0900C) derived from the dedicated lunar gravity mission GRAIL (Gravity Recovery and Interior Laboratory; Konopliv et al., 2014) was used to generate Figure a and b. The gravity anomalies were calculated using the SHTOOLS software (Wieczorek, 2014). Figure c and d are filtered gravity anomalies, computed using the “grdfft” command in the Generic Mapping Tools (GMT) software (Wessel et al., 2013). After the computation of gravity anomalies, we used ESRI ArcGIS 10.4 to prepare the maps.

The impact cratering process creates gravity anomalies in the form of a broad, circular gravity anomaly concentric to the crater. This is because the impact cratering process changes the density of the target rocks and deforms the subsurface strata. The GRAIL gravity data provide a three-dimensional perspective on impact crater and basin substructure, partially offsetting the degradation effects of superposed craters and volcanism^29^. Lunar impact craters have obvious responses to circular gravity anomalies. Therefore, high resolution gravity data would make it possible to create an additional examinations to identify specific FFCs origins, such as magmatic intrusion and sill creation or viscous relaxation. On the Petavius crater floor, north and south, there are a few dark patches of pyroclasts (dark mantle deposits) that are evidence of explosive volcanic activity. We investigated the GRAIL gravity anomalies to understand the dark mantle deposits on the crater bottom and the volcanic history of the Petavius crater. Key structural elements of impact craters/basins and floor-fractured craters are resolved by GRAIL gravity anomalies^20,38^. The free air gravity anomaly map of the Petavius crater (Fig. 7a), ranging from − 288 to 241 mGal, shows significant positive anomalies at the rim of the crater, low negative anomalies at the floor, and positive anomalies on the central peak. The topography changes in the Petavius crater are reflected in the free-air gravity anomaly (Fig. 7a); we observed the extent of the rim, fractures, and the high anomaly at the center of the crater, indicating the higher density body concerning the surroundings. This indicates the high-density body intrusion like Sill/Dyke. The Bouguer gravity anomalies (Fig. 7b) were created using the crustal density of 2.55 g/cm^3^. They show positive gravity anomalies (15–28 mGal) at the central peak and southwestern part of the crater, as well as broad, low negative gravity anomalies (− 210 to − 100 mGal) in the northern part of the crater, especially in the northern part of the rim.

Furthermore, some of the observed positive anomalies may result from the Bouguer gravity signature being influenced by pre-existing subsurface density structure and post-volcanic events (such as magmatism). The observed high positive gravity anomalies in the center and SW portion of the Petavius floor indicate the magmatic intrusion, Sill/Dyke. The high positive gravity anomalies are also indications of basaltic magma intruding beneath a crater. These can cause magmatic intrusions in floor-fractured craters (FFCs), which have deformed, uplifted, and fractured floors. Jozwiak et al. (2017)^20^ showed the positive gravity anomalies order of ~ 10–30 mGal over the central part of the Petavius crater and some parts of the rim from the band-filtered Bouguer anomalies (100–600). Baker et al. (2017)^16^ also showed the positive gravity anomalies (~ 20 mGal) at the central peak of the Petavius crater, and the authors interpreted these positive gravity anomalies as conceivably linked with an underlying magmatic sill of high density.

A regional gravity field has been calculated based on the assumption of Airy isostasy and with compensation for the crust-mantle boundary. The calculated regional gravity anomalies over the Petavius crater (Fig. 7c) show the high values at the floor of the crater and lows at the western and southern portions of the ejecta blanket and outer part of the crater rim, ranging from 3 to 66 mGal. The regional gravity anomalies are associated with more profound sources and correspond to the regional trend of the Bouguer gravity anomaly. The presence of a positive signature in the regional gravity anomalies may indicate that the formation and modification of the crater influence the deeper processes. A positive Bouguer gravity anomaly within a crater floor on the Moon could be caused by the intrusion of basaltic magma beneath the crater^29^. The isostatic residual gravity anomaly map (Fig. 7d), ranging from − 247 to 23 mGal, shows the highs (~ 10–23 mGal) at the center of the floor and southwest portion of the crater, lows at the northern rim of the crater. The gravity highs show mafic igneous bodies emplaced in rift or magmatic intrusions controlled by structures, transitional crust, and uplifted material and density variations at the shallow level. The causes of these density variations may be due to variations in composition (e.g., mafic mineral) as previous studies^21^ also interpreted the central peak of the Petavius crater had rich minerals like plagioclase, olivine, and pyroxene.

### Crustal thickness and model for emplacement

**Fig. 8:**
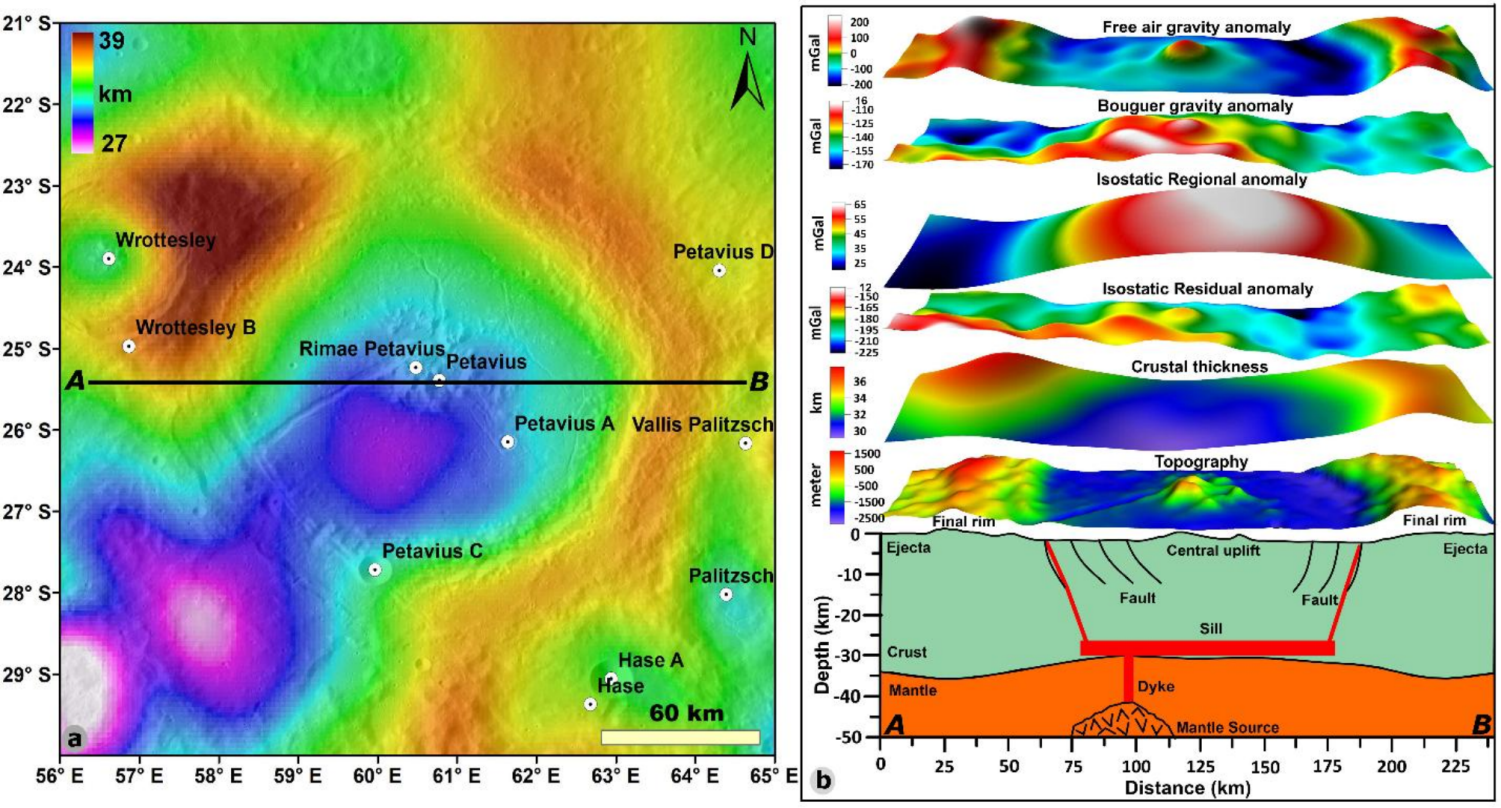
(**a**) Crustal thickness variations within the study region (GRAIL crustal thickness model-1; Wieczorek et al., 2013), (**b**) Schematic diagram along the profile AB shows the magmatic sill intrusion, formation of rille, faults, and corresponding gravity and topography anomalies. The global crustal thickness data were downloaded from the GRAIL crustal thickness archive (Model‐1 of Wieczorek et al., 2013) and extracted for the region of interest in this study (Figure a) using Generic Mapping Tools (GMT) software^40^. After extraction, we used ESRI ArcGIS 10.4 to prepare Figure a. Figure b is prepared using the Surfer 16 from Golden Software, LLC (www.goldensoftware.com).

GRAIL gravity anomalies were used to generate global crustal thickness and Moho relief models (the difference between the crust and mantle) on the Moon^39^, and the lunar community has extensively used these models to understand the subsurface structure in many case studies. The crustal thickness map of the Petavius crater (Fig. 8a) is extracted from GRAIL crustal model-1 (^39^; https://zenodo.org/record/997347#.WtIQw4huaHs). The extracted crustal thickness map varies from 27.3 to 39.5 km, the thick crust (~ 33–39.5 km) is observed at the northwest and east rims of the crater, and the thin crust (~ 27.3 to 32.5 km) is found at central floor of the crater and southwest portion of the crater (Fig. 8a). The crustal thickness map reflects the crater morphology, i.e., rim, rille, and fracture zones. We also observed thin crust along the fracture zones and rille with high Bouguer gravity anomalies. Using a W-E profile (AB in Fig. 8b), the changes in crustal thickness between the Petavius crater’s rim, floor, and outside were examined. In the subsurface cross-section along the chosen profile AB shown in Fig. 8b, we observed central mantle uplift of 1.5–2.3 km, which is in good agreement with Martinot et al. (2018)^21^. They showed that the difference between the crustal thickness and the peak material depth of origin of the Petavius crater is about + 1.6 km^21^. This is because the magmatic intrusion was primarily responsible for the volcanic activity within the crater.

The central positive gravity anomaly of the Petavius crater is due to the mantle uplift/thinning of the crust, which correlated to the other studies^12^. Although most uplifts are not confirmed as peak ring basins^16,38,39^, the Moho uplift is visible in many complex craters. According to earlier research in several case studies^38,41–43^, the Moho depression and crust thickening are thought to be the causes of the negative Bouguer gravity anomaly, the northwest and east portions of the crater having thick crust due to the negative Bouguer anomaly (Fig. 7b). Negative gravity anomalies near Petavius crater with thick crust have different causes, including lower bulk density than the nearby rocks and a fracture surface.

We study how basaltic magmatic intrusion into the crust (dikes and sills) may alter the density structure and gravity signatures of the crust surrounding the Petavius crater. The positive Bouguer gravity anomalies in the Moon’s crust have previously been interpreted by as (i) The crust-mantle boundary has positive topography (e.g.,^38^), (ii) Buried craters filled with lava (e.g.,^44,45^), (iii) Development of sills beneath floor fractured craters (e.g.,^10,20,46^), (iv) Sills that are several km thick that have penetrated the upper crust; the sill’s apparent excessive thickness is countered by the magnitude to which the intruded crust has been densified through thermal annealing and magmatic percolation (e.g.,^47^) and (v) Vertical dike complexes over a mantle diapiric magma source region (e.g.,^1^). These interpretations facilitate understanding the magmatic processes of the shallow crustal structure.The fracture system and positive gravity anomalies (Figs. 1b and 7b) found on the floor of the Petavius crater indicate that thermal annealing from the dike or sill intrusion plays a role in sealing the fractures and solidifying the nearby country rock. The crustal structure observed and interpreted (Fig. 8b) favors the possibility that sills will form at the crust-mantle boundary and rise to the surface through weak zones. We conclude that, while sill emplacement and lunar mare basalt magmatic dike should be taken into account when interpreting gravity signatures, the effects of thermal annealing and magmatic percolation are likely to be insignificant when compared to the density contrast of the solidified basaltic magmatic intrusion itself.

## Discussion

Most lunar volcanism occurred between 3.4 and 3.7 Ga, but it remained active for a considerable time (about 1.1–4.0 Ga^48–50^). Previously, the dark mantle deposits were identified in the crater’s floor and were thought to have formed by pyroclastic deposits^18,22^. A sill is a probable source of porphyritic material that would have erupted through the floor fractures into the low-lying parts of the crater floor, as well as any possible pyroclastic accumulations, as suggested by the morphometric analysis conducted on floor-fractured craters similar to the Petavius crater by Jozwiak et al. (2015)^10^. Jozwiak et al. (2017)^20^ studied the relationship between surface volcanic deposits in Petavius and positive gravity anomalies, which are related to sub-crater magmatic bodies similar to those found in the Humboldt crater.

The mafic minerals are observed along the inner walls, which have extensively excavated the basaltic units to several hundred meters within the Petavius crater. The reflective spectral signature acquired from basaltic units displays more pronounced and broader absorptions in Band I and Band II. The depth of Band-I varies within the range of ~ 4% to 18.5%, while the depth of Band-II varies within the range of ~ 2.7% to 9.6%. Furthermore, the reflective data reveals a distinct pattern characterized by more confined B-I absorptions and significantly enhanced B-II absorptions. These observations strongly suggest that High-Ca clinopyroxene predominantly influences the reflective signatures. The evident variations in BC-I and BC-II directly arise from the diverse pyroxene compositions in the region under examination. As illustrated in Fig. 4, BC-I encompasses a range from 0.93 to 1 µm.

In contrast, BC-II spans from 1.7 to 2.14 µm, indicating the coexistence of sub-calcic augite and augite compositions within the geological context of the Petavius crater^15,51^. Within the context of the BAR vs. BC-I plot (Fig. 4b), both lunar HCP and basalts consistently manifest above the mixing line that represents the blend of olivine (OL) and orthopyroxene (OPX) spectra. This behavior aligns with the notable spectral influence of HCP, as reported in the work by Cloutis and Gaffey (1991)^29^. The coexistence of HCP and LCP leads to a discernible elongation of BC-I towards longer wavelengths and a modest decrease in BAR values. Consequently, these alterations position the data points above the OL-OPX mixing line, substantiating the spectral significance of HCP in the observed spectra. A small subset of data points is situated below the Lunar OL-CPX-OPX mixing line^32^, hinting at the possible abundance of glass or plagioclase in these instances (Fig. 4b). This deviation is likely attributed to the glass or plagioclase content contributing to an elevation in the BA-I parameter, thereby leading to a downward offset in the BAR values^32^. As evident from Fig. 5, the initial stages of crystallization are marked by the presence of a high-MgO melt, likely originating from an olivine-rich mantle reservoir (Olivine-basalt). As the ambient temperature decreases and the MgO content gradually diminishes, fractional melting occurs, leading to the compositional trend of basalt to leuco basalt rock. The relatively low degree of partial melting, less than 30%, points to the absence of dissolved gases and a distinct volcanic event.

The intrusion of a magmatic body beneath overlying strata (such as a crater floor) produces initial deformation and observable surface morphologies. The surface morphologies on the Petavius crater (Fig. 1) are resultant of magmatic intrusion^5^. The surface volcanic features associated with Petavius crater intrusion evolution are categorized as surface lava flows located primarily at the crater floor-wall interface (Fig. 1). There is likely a close relationship between volcanic features and floor fractures, for example, transport of magma along faults at the intrusion periphery could lead to the observed surface flows. Morphologic studies provide evidence that floor-fractured craters are formed by the intrusion of a magmatic body beneath the crater floor^3,5,10^. This interpretation is broadly supported by recent modeling work^52^ and statistical analysis of floor-fractured crater Bouguer anomalies, which are on average more positive than those measured within complex craters^53^, consistent with the intrusion of a high density magmatic body beneath the crater floor. Floor-fractured craters formed by a sub crater magmatic intrusion would possess a high-density component in addition to the low-density breccia component. This intrusion contribution may manifest as a positive Bouguer anomaly for the crater floor as a whole or perhaps as a less negative Bouguer anomaly than would be predicted. We noted the positive gravity anomaly on the Petavius crater floor (Fig. 7). The positive gravity anomaly on the floor of the crater reflects the high density component and supports the magmatic intrusion and formation hypothesis (Fig. 8). Thus, we conclude that the volcanic features associated with the Petavius crater are related to the physical transport of magma along the fractures and onto the crater floor, which creates the positive gravity anomaly and low crustal thickness at the floor and the sill body situated at the crust-mantle interface and magma reached to the surface through fractures (Fig. 8).

Calculations of the melt composition show that the primary magmas exhibit MgO contents ranging from ~ 10.3 to ~ 24.5 wt%. Notably, these calculated primary melt compositions show that the Petavius mantle’s thermal conditions were typically warm, falling within a range of acceptable mantle temperatures. This agreement aligns with what Mallik et al. (2019)^54^ discovered. The calculated melt fractions for P1, P2, P3, P4, and P5 are 0.26, 0.24, 0.02, 0.18, and 0%, respectively. These findings overwhelmingly indicate the possibility of low-viscous volcanic eruptions in these mare units. The chemical diversity of pyroxene in basaltic rock is effectively depicted on the pyroxene quadrilateral plot, highlighting the varying proportions of Ca, Mg, and Fe components within the mineral. The chemical trajectory observed in units P1 and P2, running parallel to the Enstatite-Diopside join of the pyroxene quadrilateral, indicates the presence of augite.

**Fig. 9:**
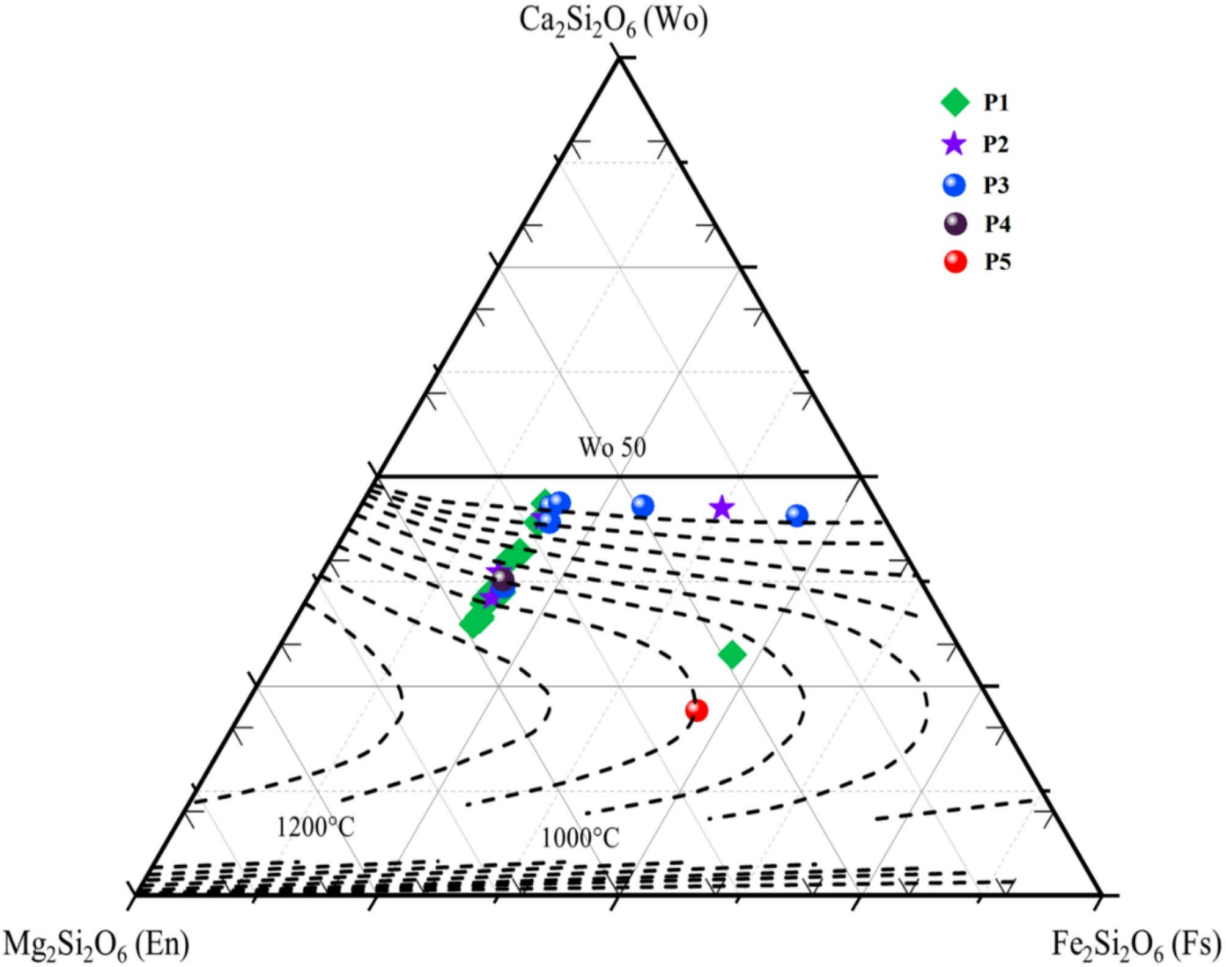
The Chemical diversity of pyroxenes of the Petavius craters depicted on the pyroxene thermometer^57^.

Crystallization initiates with compositions characterized by Mg and Ca-rich augite and transitions towards a more Ca-rich end member as the temperature decreases. The temperature range for crystallization in the P1 unit extends from around 1200 °C to 500 °C. Pyroxenes from the P3 unit display a compositional cluster near the Diopside-Hedenbergite join, as illustrated in Fig. 9. Such clustering is frequently observed in pyroxenes from rapidly cooled basalts^55^. The crystallization of augite in the P4 unit occurs at 1000 °C, deriving from the Fe rich melt. The ferroaugite compositions observed in units P1 and P5 (Fig. 9) suggest their genesis through metastable crystallization originating from a melt that experienced rapid cooling in the later stages. However, under conditions of magma cooling at a slower rate, there exists the potential for delayed quenching of the residual melt during the final stages, resulting in the crystallization of metastable pyroxene compositions that commonly exhibit instability within the forbidden region of the plot^56^. Consequently, iron-rich pyroxenes in these basalts are feasibly attributed to magma fractionation occurring within environments characterized by rapid cooling^51^. The crystallization of iron-rich pyroxene from the magma occurred at around 1100 °C, as indicated by the readings on the pyroxene thermometer^57^.

## Conclusions

This paper calculated the primary magma compositions of volcanic that occurred in Petavius crater on the Moon, revealing the lunar mantle’s thermal regime beneath this region. M^3^ data reveals the presence of feldspar mineral-plagioclase and olivine at the central peak and other mafic minerals like Low-Ca pyroxene and High-Ca Pyroxene in distinct mare units. Primary melt composition suggests that the thermal regime of the Petavius mantle was not anomalously hot (i.e., > 1500 °C) and indicates the pyroxene fractionation from the depleted peridotite melt. The central peak of the Petavius crater has potential pyroclastic deposits, which is a reasonably typical occurrence on the Moon. However, because the floor has a flat topography and slight albedo contrast, it is frequently challenging to identify mantling on dark-toned mare deposits. The trapping of low melt fraction deposits with pyroclastic indicates that explosive volcanism took place near the end of this age of volcanism, possibly due to the stalling and deformation of the last magma pulse under the surface of the Petavius crater. The process of magmatic intrusion and sill development also poses a significant geophysical restriction because it is anticipated that the dense magma body will contribute positively to the Bouguer anomaly at the crater’s central peak. The shallow crust beneath the central peak of the Petavius crater, together with the positive gravity anomalies in Bouguer and Isostatic residual, show that magmatic volcanic activity was involved in the crater’s formation and modification. Our combined analysis of composition and gravity anomalies points to magmatic intrusion and sill formation beneath the Petavius crater floor as well as a source that is likely responsible for porphyritic mare material that erupted through the crater floor’s floor fractures into low-lying areas.

## Methods

Individual data strips acquired by the Moon Mineralogy Mapper (M^3^) instrument were subjected to geo-referencing processes to yield a cohesive mosaic encompassing the specified region of interest. In our workflow, we harnessed the capabilities of ENVI (Environment for Visualizing Images) to process data and produce the desired results. The M^3^ hyperspectral data depict the mineralogy. Mafic lunar minerals, exemplified by pyroxenes, conspicuously exhibit dual absorption features within the spectral realms around 1 µm and 2 µm. Importantly, the precise location of these absorption band centers undergoes a discernible redshift, moving toward longer wavelengths, in correlation with an augmented presence of iron (Fe) or calcium (Ca) within the mineral matrix^58^. Orthopyroxene and pigeonite (Low-Ca Clinopyroxene), scientifically referred to as Low-Calcium Pyroxene (LCP), prominently showcase their absorption maxima within the spectral regions spanning 0.90–0.97 µm and 1.8–2.1 µm. Conversely, clinopyroxene (e.g., augite), denoted as High-Calcium Pyroxene or High-Ca Clinopyroxene (HCP), exhibits its peak absorptions proximal to the wavelength intervals of 0.91–1.06 µm and 1.97–2.3 µm. These distinct absorption profiles serve as discriminative indicators for unequivocally discerning and classifying these two pyroxene mineral variants^29,59^. Olivine has single board absorption at 1.05 µm. Plagioclase has single aborption at 1.25 µm^44^. Discriminating between different lunar lithologies is facilitated by the comprehensive examination of both the band centers and the amplitude of reflectance spectra. Obtaining samples from optically immature, freshly exposed regions is crucial to examine the mineralogical variability without interference from space weathering effects associated with optical maturity. Optical maturity can lead to apparent shifts in the band center positions^60^. Consequently, spectra were acquired from the well-exposed sections of the crater walls. The average spectra were derived by identifying the maxima values from four pixels within only fresh exposure.

### Compositional analysis

The band parameter calculations were done following the procedures defined by^15,51,61,62^. Foremost, the spectra were smoothed as described by^19^, and the quantification of Band Center (BC), Band Depth (BD), and Band Area (BA) metrics involved a methodical examination. The process is discussed in detail in the supplementary file S. These parameter values created the Band I vs. Band II, Band I vs. BAR (Band Area Ratio), and pyroxene quadrilateral graph. We utilized the COMAGMAT application^33^ to calculate the primary melt makeup and mantle potential temperatures of Petavius mare rocks from the elemental oxide abundance maps. The COMAGMAT model is a numerical framework used to simulate crystallization dynamics and associated magmatic processes in volcanic and plutonic environments across varying depths and oxidation states^63^. The model operates on an algorithm that reproduces equilibrium and fractional crystallization in multi-saturated melts^63^. It utilizes phase equilibrium equations, functioning as thermometric tools, to accurately predict crystallization temperatures (within 10–15 °C) and phase compositions (with 1–3 mol% accuracy)^63^. The oxygen fugacity was set at the iron-wüstite buffer, relevant for mare basalts and lunar igneous glasses^64,65^, with key parameters sourced from Sakai et al. (2014)^66^. The aim was to ascertain the likely thermal conditions following the methodology established by Herzberg and Asimow (2015)^67^.

### Gravity, isostatic regional, and residual anomalies

Bouguer and Free-air gravity anomalies are computed from JGGRX_0900C gravity model up to 660 degree using SHTOOLS. The data for the Petavius region are then re-projected using grdproject in Generic Mapping Tools using a Mercator projection with origin at 0E, 0N. The study area is only 30° S to 21° S; therefore, there is minimal distortion in the shape of the feature. The Bouguer gravity anomalies over the Petavius crater are calculated using the crustal density of 2.55 g/cm^3^ between 2 and 660 degrees to eliminate high-frequency noise. We computed Bouguer gravity anomalies using the method put forth by Wieczorek and Phillips in 1998, which was then applied in other studies^68,69^. Although the Bouguer reduction procedure eliminates the gravitational influence of topography, it does not consider the effect of the compensating mass at depth; therefore, there is still a correlation with topography. It would be helpful to adjust to take this effect out to improve the signal caused by intra-crustal density contrasts.

The topography-induced regional gravity gradients were removed using a variety of techniques. These gradients can be distinguished from and eliminated by wavelength filtering since their wavelengths tend to be lengthy. The drawback of such methods is that they might eliminate long-wavelength signals from intra-crustal sources of geologic significance. Alternatively, a correction may be made using a smoothed-out representation of the topography. A model of isostatic compensation of the regional topographic load is produced due to suppressing high-frequency topographic variations. It is possible to create a local gravitational field that correlates with topography and can be used as a correction by developing a smoothing function proportional to the anticipated compensation depth and using the proper factor for conversion from a topographic load to a gravitational field. Consequently, isostatic adjustment is best suited to improve the changes in crustal density. Isostatic compensation, presented in a model to account for isostatic anomalies, is the flexural adjustment of the lithosphere, growth in topography, or existence of low-density roots. It is a process in which lateral movements compensate for lateral transport of the surface in a sub-crustal layer. Because the crust’s composition, temperature, and porosity vary greatly, you must be concerned with how crust density varies from location to location.

According to several studies^43,70^, the topography of the Moon may feature isostatically compensating roots. As a result, we additionally consider gravity anomalies that comprise contributions from crustal roots. It has been determined through studies based on Clementine^43^ and GRAIL^71^ data that Airy isostasy, not Pratt isostasy, is possible to be the predominant isostatic compensation mechanism on the Moon. In this study, we employed the Airy model to compute isostatic gravity anomalies, where the presence of the crustal root, whose thickness varies in response to the elevation of the topography, compensates for the topographic mass. The topographic density in the Airy model was 2.55 g/cm^3^, the crustal thickness was 36 km, and the density contract across the base of the model crust was 0.65 g/cm^3^, according to GRAIL observations^39^ and Apollo seismic data^72^. Elkins-Tanton et al. (2011)^73^ calculated mineral densities as a function of depth along with theoretical models of lunar magma ocean solidification and successive mantle overturn predicted an upper mantle density that monotonically increased with depth between 3.0 and 3.2 g/cm^3^, with a distinct jump in density at 700 km depth.

## Electronic supplementary material

Below is the link to the electronic supplementary material.

Supplementary Material 1

## Data Availability

All the data sets used in this study area are publicly available. The PDS Geoscience Node, Lunar Orbital Data Explorer (http://ode.rsl.wustl.edu/) provides access to the M^3^, LRO-LOLA digital elevation model, and LRO WAC data. The GRAIL crustal thickness model-1 was downloaded from https://zenodo.org/record/997347#.WtIQw4huaHs. The gravity model GL0900C was taken from the Geosciences Node of Planetary Data System (http://pds-geosciences.wustl.edu/missions/grail/default.htm) for this investigation. However, the generated datasets from this study are available with the corresponding author and can be freely accessed.

## References

[1] Head, J. W. & Wilson, L. Lunar mare volcanism: Stratigraphy, eruption conditions, and the evolution of secondary crusts. *Geochim. Cosmochim. Acta* **56**, 2155–2175 (1992).

[2] Spudis, P. D., McGovern, P. J. & Kiefer, W. S. Large shield volcanoes on the Moon. *JGR Planets* **118**, 1063–1081 (2013).

[3] Jozwiak, L. M., Head, J. W., Zuber, M. T., Smith, D. E. & Neumann, G. A. Lunar floor-fractured craters: Classification, distribution, origin and implications for magmatism and shallow crustal structure: Floor-fractured crater distribution and origin. *J. Geophys. Res.* **117**, (2012).

[4] Sruthi, U. & Senthil Kumar, P. Volcanism on farside of the Moon: New evidence from antoniadi in south pole aitken basin. *Icarus* **242**, 249–268 (2014).

[5] Satyakumar, A. V. & Patel, S. Morphological and chronological mapping of Petavius crater, nearside of the Moon. *Adv. Space Res.* **74**, 6124. 10.1016/j.asr.2024.08.074 (2024).

[6] Croft, S. K. Lunar crater volumes-Interpretation by models of impact cratering and upper crustal structure. *Lunar Planetary Sci. Conf.* **9**, 3711–3733 (1978).

[7] Young, R. A. & Brennan, W. J. *Selected aspects of lunar mare geology from apollo orbital photography*. https://ntrs.nasa.gov/citations/19760009914 (1976).

[8] Michaut, C., Pinel, V. & Maccaferri, F. Magma ascent at floor-fractured craters diagnoses the lithospheric stress state on the Moon. *Earth Planet. Sci. Lett.* **530**, 115889 (2020).

[9] Pinel, V. & Jaupart, C. The effect of edifice load on magma ascent beneath a volcano. *Philos. Trans. Royal Soc. Lond. Ser. A Math. Phys. Eng. Sci.* **358**, 1515–1532 (2000).

[10] Jozwiak, L. M., Head, J. W. & Wilson, L. Lunar floor-fractured craters as magmatic intrusions: Geometry, modes of emplacement, associated tectonic and volcanic features, and implications for gravity anomalies. *Icarus* **248**, 424–447 (2015).

[11] Schultz, P. H. Floor-fractured lunar craters. *The Moon* **15**, 241–273 (1976).

[12] Walwer, D., Michaut, C., Pinel, V. & Adda-Bedia, M. Magma ascent and emplacement below floor fractured craters on the Moon from floor uplift and fracture length. *Phys. Earth Planetary Interiors* **312**, 106658 (2021).

[13] Hawke, B. R. et al. An investigation of cryptomare and pyroclastic deposits in the gassendi region of the Moon. 1894 (2013).

[14] Hawke, B. R. et al. Cryptomare, lava lakes, and pyroclastic deposits in the gassendi region of the Moon: Final results. 1310 (2015).

[15] Purohit, A. N., Patel, S. M., Thaker, A. D. & Solanki, P. M. Compositional and morphological analysis of Gassendi crater. *J. Earth Syst. Sci.* **130**, 57 (2021).

[16] Baker, D. M. H. et al. GRAIL gravity observations of the transition from complex crater to peak-ring basin on the Moon: Implications for crustal structure and impact basin formation. *Icarus* **292**, 54–73 (2017).

[17] Chauhan, M., Tiwari, P. S. & Chauhan, P. Geological investigation of petavius crater, Moon using high-resolution datasets from recent lunar missions. 1843 (2021).

[18] Gaddis, L. R., Staid, M. I., Tyburczy, J. A., Hawke, B. R. & Petro, N. E. Compositional analyses of lunar pyroclastic deposits. *Icarus* **161**, 262–280 (2003).

[19] Gustafson, J. O., Gaddis, L. R., Bell, J. F. & Gustafson, J. A. An investigation of potential pyroclastic deposits on the southeast limb of the Moon. *Icarus* **349**, 113828 (2020).

[20] Jozwiak, L. M., Head Iii, J. W., Neumann, G. A. & Wilson, L. Observational constraints on the identification of shallow lunar magmatism: Insights from floor-fractured craters. *Icarus* **283**, 224–231 (2017).

[21] Martinot, M. et al. Mineralogical diversity and geology of humboldt crater derived using Moon mineralogy mapper data. *J. Geophys. Res.: Planets* **123**, 612–629 (2018). 29938148 10.1002/2017JE005435 PMC5993347

[22] Wilhelms, D. E. & El-Baz, F. Geologic map of the east side of the Moon. (1977).

[23] Thomas, R. J., Rothery, D. A., Conway, S. J. & Anand, M. Explosive volcanism in complex impact craters on mercury and the Moon: Influence of tectonic regime on depth of magmatic intrusion. *Earth Planetary Sci. Lett.* **431**, 164–172 (2015).

[24] Michaut, C. & Pinel, V. Magma ascent and eruption triggered by cratering on the Moon: Magma ascent below impact craters. *Geophys. Res. Lett.* **45**, 6408–6416 (2018).

[25] Ma, M. et al. Global estimates of lunar surface chemistry derived from LRO diviner data. *Icarus* **371**, 114697 (2022).

[26] Zhang, L. et al. New maps of major oxides and Mg # of the lunar surface from additional geochemical data of Chang’E-5 samples and KAGUYA multiband imager data. *Icarus* **397**, 115505 (2023).

[27] Ma, M. et al. High alumina basalts identification and their feature analysis in Mare Fecunditatis. *Icarus* **407**, 115464. 10.1016/j.icarus.2023.115464 (2023).

[28] Kramer, G. Y., Jolliff, B. L. & Neal, C. R. Searching for high alumina mare basalts using Clementine UVVIS and Lunar Prospector GRS data: Mare Fecunditatis and Mare Imbrium. *Icarus* **198**, 7–18 (2008).

[29] Cloutis, E. A. & Gaffey, M. J. Pyroxene spectroscopy revisited: Spectral-compositional correlations and relationship to geothermometry. *J. Geophys. Res.* **96**, 22809 (1991).

[30] Klima, R. L., Dyar, M. D. & Pieters, C. M. Near-infrared spectra of clinopyroxenes: Effects of calcium content and crystal structure: Near-infrared spectra of clinopyroxenes. *Meteoritics Planetary Sci.* **46**, 379–395 (2011).

[31] Cloutis, E. A., Gaffey, M. J., Jackowski, T. L. & Reed, K. L. Calibrations of phase abundance, composition, and particle size distribution for olivine-orthopyroxene mixtures from reflectance spectra. *J. Geophys. Res.* **91**, 11641 (1986).

[32] Zhang, X. & Cloutis, E. Near-infrared spectra of lunar ferrous mineral mixtures. *Earth Space Sci.* **8**, e2020EA001153 (2021).

[33] Ariskin, A. A. & Barmina, G. S. COMAGMAT: Development of a magma crystallization model and its petrological applications. *Geochemistry International* **42**, S1–S157 (2004).

[34] Herzberg, C. & O’Hara, M. J. Plume-associated ultramafic magmas of phanerozoic age. *J. Petrol.* **43**, 1857–1883 (2002).

[35] Cordell, D., Naif, S., Troch, J. & Huber, C. Constraining magma reservoir conditions by integrating thermodynamic petrological models and bulk resistivity from magnetotellurics. *Geochem. Geophys. Geosyst.* **23**, e2022GC010455 (2022).

[36] Lemelin, M. et al. The compositions of the lunar crust and upper mantle: Spectral analysis of the inner rings of lunar impact basins. *Planetary Space Sci.* **165**, 230–243 (2019).

[37] *Igneous rocks: A classification and glossary of terms: Recommendations of the international union of geological sciences subcommission on the systematics of igneous rocks*. (Cambridge University Press, Cambridge, 2002). 10.1017/CBO9780511535581.

[38] Neumann, G. A. et al. Lunar impact basins revealed by Gravity Recovery and Interior laboratory measurements. *Sci. Adv.* **1**, e1500852 (2015). 26601317 10.1126/sciadv.1500852 PMC4646831

[39] Wieczorek, M. A. et al. The crust of the Moon as seen by GRAIL. *Science* **339**, 671–675 (2013). 23223394 10.1126/science.1231530 PMC6693503

[40] Wessel, P., Smith, W. H. F., Scharroo, R., Luis, J. & Wobbe, F. Generic mapping tools: Improved version released. *Eos, Trans. Am. Geophys. Union* **94**, 409–410 (2013).

[41] Melosh, H. J. et al. The origin of lunar mascon basins. *Science* **340**, 1552–1555 (2013). 23722426 10.1126/science.1235768

[42] Neumann, G. A., Zuber, M. T., Smith, D. E. & Lemoine, F. G. The lunar crust: Global structure and signature of major basins. *J. Geophys. Res.* **101**, 16841–16863 (1996).

[43] Wieczorek, M. A. & Phillips, R. J. Potential anomalies on a sphere: Applications to the thickness of the lunar crust. *J. Geophys. Res.* **103**, 1715–1724 (1998).

[44] Evans, A. J. et al. Reexamination of early lunar chronology with GRAIL data: Terranes, basins, and impact fluxes. *JGR Planets* **123**, 1596–1617 (2018).

[45] Huang, Q., Xiao, Z. & Xiao, L. Subsurface structures of large volcanic complexes on the nearside of the Moon: A view from GRAIL gravity. *Icarus* **243**, 48–57 (2014).

[46] Wilson, L. & Head, J. W. Lunar floor-fractured craters: Modes of dike and sill emplacement and implications of gas production and intrusion cooling on surface morphology and structure. *Icarus* **305**, 105–122 (2018).

[47] Kiefer, W. S. Gravity constraints on the subsurface structure of the Marius Hills: The magmatic plumbing of the largest lunar volcanic dome complex. *JGR Planets* **118**, 733–745 (2013).

[48] Head, J. W. Lunar volcanism in space and time. *Rev. Geophys.* **14**, 265 (1976).

[49] Hiesinger, H., Head, J. W., Wolf, U., Jaumann, R. & Neukum, G. Ages and stratigraphy of lunar mare basalts: A synthesis. In *Recent Advances and Current Research Issues in Lunar Stratigraphy* (Geological Society of America, 2011). 10.1130/2011.2477(01).

[50] Hiesinger, H., Head, J. W., Wolf, U., Jaumann, R. & Neukum, G. Ages and stratigraphy of mare basalts in oceanus procellarum, mare nubium, mare cognitum, and mare insularum. *J. Geophys. Res.* **108**, 2002JE001985 (2003).

[51] Patel, S. M., Harish, P. D., Solanki, P. M. & El-Maarry, M. R. Hybrid volcanic episodes within the orientale basin, Moon. *Remote Sens.* **15**, 1801 (2023).

[52] Thorey, C. & Michaut, C. A model for the dynamics of crater-centered intrusion: Application to lunar floor-fractured craters. *J. Geophys. Res. Planets* **119**, 286–312 (2014).

[53] Thorey, C., Michaut, C. & Wieczorek, M. Gravitational signatures of lunar floor-fractured craters. *Earth Planetary Sci. Lett.* **424**, 269–279 (2015).

[54] Mallik, A., Ejaz, T., Shcheka, S. & Garapic, G. A petrologic study on the effect of mantle overturn: Implications for evolution of the lunar interior. *Geochimica et Cosmochimica Acta* **250**, 238–250 (2019).

[55] Papike, J. J., Hodges, F. N., Bence, A. E., Cameron, M. & Rhodes, J. M. Mare basalts: Crystal chemistry, mineralogy, and petrology. *Rev. Geophys.* **14**, 475 (1976).

[56] Papike, J. J., Bence, A. E., Brown, G. E., Prewitt, C. T. & Wu, C. H. Apollo 12 clinopyroxenes: Exsolution and epitaxy. *Earth Planetary Sci. Lett.* **10**, 307–315 (1971).

[57] Lindsley, D. H. & Andersen, D. J. A two-pyroxene thermometer. *J. Geophys. Res.* **88**, A887 (1983).

[58] Klima, R. L., Pieters, C. M. & Dyar, M. D. Spectroscopy of synthetic Mg-Fe pyroxenes I: Spin-allowed and spin-forbidden crystal field bands in the visible and near-infrared. *Meteoritics Planetary Sci.* **42**, 235–253 (2007).

[59] Adams, J. B. Visible and near-infrared diffuse reflectance spectra of pyroxenes as applied to remote sensing of solid objects in the solar system. *J. Geophys. Res.* **79**, 4829–4836 (1974).

[60] Clark, R. N. & Roush, T. L. Reflectance spectroscopy: Quantitative analysis techniques for remote sensing applications. *J. Geophys. Res.* **89**, 6329–6340 (1984).

[61] Horgan, B. H. N., Cloutis, E. A., Mann, P. & Bell, J. F. Near-infrared spectra of ferrous mineral mixtures and methods for their identification in planetary surface spectra. *Icarus* **234**, 132–154 (2014).

[62] Kaur, P., Bhattacharya, S., Chauhan, P., Ajai, & KiranKumar, A. S. Mineralogy of mare serenitatis on the near side of the Moon based on chandrayaan-1 Moon mineralogy mapper (M^3^) OBSERVATIONS. *Icarus* **222**, 137–148 (2013).

[63] Ariskin, A. A. Phase equilibria modeling in igneous petrology: use of COMAGMAT model for simulating fractionation of ferro-basaltic magmas and the genesis of high-alumina basalt. *J. Volcanol. Geothermal Res.* **90**, 115–162 (1999).

[64] Sato, M., Hickling, N. L. & McLane, J. E. Oxygen fugacity values of Apollo 12, 14, and 15 lunar samples and reduced state of lunar magmas. *Lunar Planetary Sci. Conf. Proc.* **4**, 1061 (1973).

[65] Wellman, T. R. Gaseous species in equilibrium with the apollo II holocrystalline rocks during their crystallization. *Nature* **225**, 716–717 (1970). 16056711 10.1038/225716a0

[66] Sakai, R., Nagahara, H., Ozawa, K. & Tachibana, S. Composition of the lunar magma ocean constrained by the conditions for the crust formation. *Icarus* **229**, 45–56 (2014).

[67] Herzberg, C. & Asimow, P. D. PRIMELT3 MEGA.XLSM software for primary magma calculation: Peridotite primary magma MgO contents from the liquidus to the solidus. *Geochem. Geophys. Geosyst.* **16**, 563–578 (2015).

[68] Konopliv, A. S. et al. High-resolution lunar gravity fields from the GRAIL Primary and Extended Missions. *Geophys. Res. Lett.* **41**, 1452–1458 (2014).

[69] Satya Kumar, A. V., Rajasekhar, R. P. & Tiwari, V. M. Gravity anomalies and crustal structure of the Lunar far side highlands. *Planetary Space Sci.* **163**, 106–113 (2018).

[70] Zuber, M. T., Smith, D. E., Lemoine, F. G. & Neumann, G. A. The shape and internal structure of the Moon from the clementine mission. *Science* **266**, 1839–1843 (1994). 17737077 10.1126/science.266.5192.1839

[71] Sori, M. M. et al. Isostatic compensation of the lunar highlands. *JGR Planets* **123**, 646–665 (2018).

[72] Khan, A. & Mosegaard, K. An inquiry into the lunar interior: A nonlinear inversion of the Apollo lunar seismic data. *J.-Geophys.-Res.* **107**, (2002).

[73] Elkins-Tanton, L. T., Burgess, S. & Yin, Q.-Z. The lunar magma ocean: Reconciling the solidification process with lunar petrology and geochronology. *Earth Planetary Sci. Lett.* **304**, 326–336 (2011).

