# Supplementary material for: Volcanic processes within the Petavius crater, nearside of the Moon
Source: Sci Rep. 2025 Mar 24;15:10176. doi: 10.1038/s41598-025-95132-5 (PMC11933338; doi:10.1038/s41598-025-95132-5)
Supplement: Supplementary file 1 — Supplementary Material 1 [file 41598_2025_95132_MOESM1_ESM.docx]

Supplementary information for

**Volcanic processes within the Petavius crater, nearside of the Moon”**

A.V. Satyakumar^1^, Shreekumari Patel^2*^, Deep Dixit Patel^3^

^1^CSIR-National Geophysical Research Institute (CSIR-NGRI), Hyderabad, 500007, India.
^2^Space and Planetary Science Group, Department of Earth Sciences, Khalifa University, Abu Dhabi, United Arab Emirates.

^3^Department of Earth Sciences, University of Western Ontario, Canada.

^*^Corresponding author: ipatelshree@gmail.com

**Methodology of Moon Mineralogy Mapper (M^3^)**

The spectral data is integrated representation of the spectral signatures from diverse elements present on the investigated surface. The spectral data is acquired from an impact craters (~1 km) with the aim of sampling optically immature or minimally weathered material, and thereby minimizing the effects of space weathering and avoiding attenuation of diagnostic absorption characteristics ^1–3^. In the spectral analysis, we identified various minerals by calculating specific features such as band center (BC), band area (BA), band depth (BD), and band area ratio (BAR), along with examining the board spectral profile. The band parameter BA is effective in emphasizing the presence of olivine, the ratio between BA1 and BA 2 is particularly useful in highlighting spinel, and the BC parameter aids in distinguishing among diverse pyroxene groups ^4^.

The presence of pyroxene will potentially mask the effects of other mixed minerals like plagioclase and/or olivine in the reflected spectra ^5^. However, coexistence of different minerals may induce a spectral gradient and cause a displacement in the apparent minima of Band I (around 1 µm) and Band II (around 2 µm). Scattering that varies with wavelength enhances the continuum slope of the spectra, leading to minimal band minima in the prominent absorption bands at 1 µm and 2 µm ^5^. Continuum removal must be applied to the spectra to minimize the effects of the continuum slope on the prominent absorption bands prior to the analysis of spectra parameters ^5^. In the continuum removal procedure, inflection point positioned adjacent to the absorption bands were employed to delineate a tangent line, facilitating the determination of the continuum slope for each individual spectrum analysed ^6^. The M^3^ data manifest a residual thermal effect ^7^, characterised by persistent rise in reflectance beyond 2 µm, thereby complicating the continuum removal procedure. Previous research has implemented varying terminal points of the spectra during the process of continuum removal, with some studies designating 2.5 µm as the endpoint (with instance, ^8–10^), while others have set it at 2.6 µm ^11^. ^9^ calculated spectral parameters, asserting that the most effective endpoints for the M3 data are located at 2.497 µm during the continuum removal process. The parameters BC, BA, and BD for each reflectance spectra are computed following the methodologies outlined in the studies by Cloutis et al. (1986) and Gaffey et al. (2002). A fourth-order polynomial function is applied to fit the lowest points of the absorption bands after the removal of continuum slope. BC parameter is determined by computing the lowest point on this fitted polynomial curve. We iteratively determined the error associated with BC values. BD measures the extent of absorption within the spectral feature in relation to the continuum level. The BA is computed as the area enclosed between the continuum line and spectral data points of the absorption band. The BAR is calculated by taking the area under Band II and dividing it by the area under Band I in a spectral analysis. BAR values are employed to discern the relative preponderance of olivine and pyroxenes within the spectral data. A higher BAR value implies significant presence of pyroxenes, whereas lower values suggest the existence of a mixed olivine phase in the reflectance spectra ^12^. The influence of olivine in the spectral data was eliminated by applying the calibration methods proposed by Cloutis et al. (1986) and Gaffey et al. (2002). A correction factor was introduced to the BC I to offset the potential influence of olivine in the spectral data prior to identifying the composition of pyroxene. It was measured by computing the shift in the BC using the calibration between the BAR and BC I. The calculated shift is applied for adjustment of olivine in the BC I. The BC I (adjusted olivine value) and BC II values are utilized to calculate the molar quantities of Ca, Fe and Mg in pyroxenes. This computation is based on series of formulas provided by Gaffey et al. (2002).


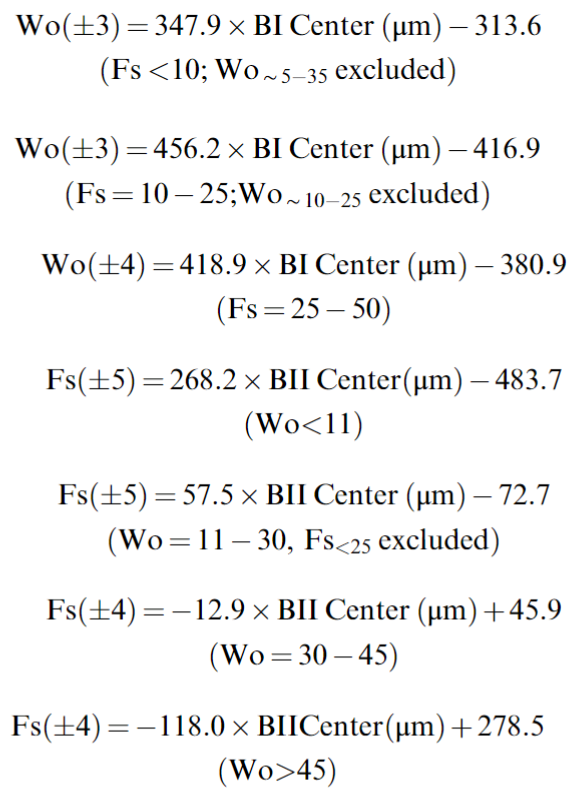


The molar concentrations of Ca, Fe and Mg are depicted on a pyroxene quadrilateral diagram to analyze the relative distribution of these compositions. The evolution of basaltic magma crystallization was inferred by examining the trend in pyroxene composition observed in the quadrilateral graph, as described by Lindsley and Andersen (1983). This technique, while powerful, may overlook microscopic features such as exsolution lamellae, especially if they are smaller than the instrument’s spatial resolution. Furthermore, the pyroxene thermometer, which estimates formation temperatures based on pyroxene composition, may not fully account for the effects of subsolidus exsolution (Lindsley and Andersen, 1983).

The list of M^3^ data strip used for the study are following:

| Strip Name | Date of Acquisition | Orbit | Period |
| --- | --- | --- | --- |
| M3G20090728T130752 | 28-07-2009 | 200 Km | Cold |
| M3G20090728T171132 | 28-07-2009 | 200 Km | Cold |
| M3G20090728T214215 | 28-07-2009 | 200 Km | Cold |

**Table S1: Band Parameters of the mare units.**

**Band center, band depth and band area measurements of locations in the mare region.**

| P1 | X Co-ordinates | Y Co-ordinates | Band-I center (µm) | Stdev(BC-I) | Band-I center (µm) corrected | B-I Area | Stdev(BA-I) | Band-II center (µm) | Stdev(BC-II) | B-II Area | Stdev(BA-II) | BAR | B-I Strength | B-II Strength | Displacement | |
| --- | --- | --- | --- | --- | --- | --- | --- | --- | --- | --- | --- | --- | --- | --- | --- | --- |
| Roi 1 | 60.39582 | -23.363 | 1.0034 | 0.0054 | 0.992736 | 41.499 | 0.46926 | 2.09174 | 0.00631 | 58.50086 | 0.99702 | 1.409693 | 5.732 | 4 |  | 0.01066396 |
| Roi 2 | 60.42145 | -23.3732 | 0.9889 | 0.0016 | 0.978082 | 44.255 | 0.6965 | 2.08632 | 0.0052 | 55.7454 | 1.45033 | 1.259641 | 10.1 | 6.5 |  | 0.01081766 |
| Roi 3 | 60.47784 | -23.363 | 1.007 | 0.0018 | 0.99173 | 55.097 | 0.45 | 2.129 | 0.0075 | 44.9024 | 0.836 | 0.81497 | 11.15 | 5.22 |  | 0.015270147 |
| Roi 4 | 60.47271 | -23.4193 | 1.001 | 0.0017 | 0.990311 | 48.24 | 0.41 | 2.119 | 0.0059 | 51.758 | 0.79141 | 1.072927 | 6.4 | 3.8 |  | 0.0106894 |
| Roi 5 | 60.52397 | -23.4142 | 1.002 | 0.00221 | 0.986925 | 50.092 | 0.7767 | 2.092 | 0.00778 | 49.9078 | 1.4647 | 0.996323 | 8.5 | 4.8 |  | 0.015075053 |
| Roi 6 | 60.5496 | -23.4347 | 1.004 | 0.00195 | 0.993342 | 48.839 | 0.42036 | 2.111 | 0.00656 | 51.16 | 0.78936 | 1.047523 | 6.108 | 3.557 |  | 0.0106576 |
| Roi 7 | 60.59574 | -23.4398 | 1.017 | 0.0028 | 1.001308 | 52.48 | 0.4892 | 2.127 | 0.0106 | 47.52 | 0.95402 | 0.905488 | 8.09 | 4.296 |  | 0.015692315 |
| Roi 8 | 60.55985 | -23.3168 | 1.022 | 0.0038 | 1.011533 | 45.035 | 1.123 | 2.064 | 0.00724 | 54.965 | 1.895 | 1.220495 | 7.2 | 5.5 |  | 0.0104668 |
| Roi 9 | 60.73414 | -23.3066 | 1.004 | 0.0036 | 0.993342 | 39.499 | 0.9964 | 2.033 | 0.011 | 60.501 | 2.378 | 1.53171 | 6.05 | 4.14 |  | 0.0106576 |
| Roi 10 | 60.61111 | -23.4142 | 1.002 | 0.0027 | 0.986925 | 52.92 | 0.749 | 2.099 | 0.011 | 47.082 | 1.405 | 0.889683 | 6.92 | 3.47 |  | 0.015075053 |
| Roi 11 | 60.71876 | -23.404 | 1.014 | 0.0037 | 1.003448 | 49.27 | 2.1677 | 2.069 | 0.0115 | 50.7269 | 3.889 | 1.02957 | 11.66 | 7.128 |  | 0.0105516 |
| Roi 12 | 60.99044 | -23.4193 | 1.0142 | 0.0037 | 1.003651 | 49.513 | 2.1677 | 2.069 | 0.0115 | 50.487 | 3.86 | 1.019672 | 11.66 | 7.093 |  | 0.01054948 |
| Roi 13 | 61.36464 | -23.6551 | 1 | 0.0029 | 0.985 | 53.12 | 1.414 | 2.113 | 0.0142 | 46.8847 | 2.912 | 0.882619 | 14.231 | 6.246 |  | 0.015 |
| Roi 14 | 61.59018 | -23.7833 | 0.997 | 0.00233 | 0.986268 | 35.88679 | 0.6 | 2.0855 | 0.0062 | 64.113 | 1.35 | 1.786535 | 6.529 | 5.429 |  | 0.0107318 |
| Roi 15 | 61.54917 | -23.6603 | 1.001 | 0.00177 | 0.990311 | 45.487 | 0.4498 | 2.103 | 0.0063 | 54.513 | 0.947 | 1.19843 | 7.014 | 4.15 |  | 0.0106894 |
| Roi 16 | 61.71833 | -23.65 | 1.006 | 0.0027 | 0.995364 | 46.08 | 1.471 | 2.073 | 0.00772 | 53.92 | 2.684 | 1.170139 | 10.305 | 7.085 |  | 0.0106364 |
| Roi 17 | 60.79565 | -23.4552 | 1.027 | 0.0029 | 1.016586 | 46.186 | 0.597 | 2.095 | 0.09267 | 53.814 | 1.1558 | 1.165158 | 5.69 | 3.55 |  | 0.0104138 |
| Roi 18 | 61.42102 | -23.6705 | 1.002 | 0.0023 | 0.991321 | 48.49 | 0.9413 | 2.101 | 0.0086 | 51.5 | 1.875 | 1.062075 | 11.22 | 6.21 |  | 0.0106788 |
| P2 |  |  | Band-I center (µm) | Stdev(BC-I) | Band-I center (µm) corrected | B-I Area | Stdev(BA-I) | Band-II center (µm) | Stdev(BC-II) | B-II Area | Stdev(BA-II) | BAR | B-I Strength | B-II Strength | Displacement | |
| Roi 1 | 61.35951 | -24.26 | 1.014 | 0.00287 | 0.998439 | 60.92 | 1.2123 | 2.142 | 0.0127 | 39.074 | 2.021 | 0.641399 | 12.466 | 5.116 |  | 0.015561187 |
| Roi 2 | 61.33901 | -24.3113 | 1.023 | 0.00366 | 1.012544 | 41.22 | 0.9629 | 2.032 | 0.012012 | 58.78 | 2.23 | 1.426007 | 5.26 | 3.483 |  | 0.0104562 |
| Roi 3 | 61.3185 | -24.3523 | 1.03 | 0.00386 | 1.019618 | 41.787 | 0.9842 | 2.042 | 0.1036 | 58.213 | 1.936 | 1.393089 | 5.393 | 3.999 |  | 0.010382 |
| Roi 4 | 61.41846 | -24.4266 | 1.007 | 0.0038 | 0.99173 | 54.154 | 2.504 | 2.078 | 0.012022 | 45.846 | 4.314 | 0.846586 | 18.48 | 9.58 |  | 0.015270147 |
| P3 |  |  | Band-I center (µm) | Stdev(BC-I) | Band-I center (µm) corrected | B-I Area | Stdev(BA-I) | Band-II center (µm) | Stdev(BC-II) | B-II Area | Stdev(BA-II) | BAR | B-I Strength | B-II Strength | Displacement | |
| Roi 1 | 60.86741 | -27.0896 | 1.0321 | 0.004301 | 1.015689 | 52.94349 | 1.36937 | 2.034 | 0.02109 | 47.05651 | 3.029 | 0.888806 | 6.285 | 2.697 |  | 0.016410593 |
| Roi 2 | 60.82641 | -27.1665 | 1.027869 | 0.002926 | 1.017464 | 48.20193 | 0.9078 | 1.97257 | 0.01493 | 51.79807 | 2.3895 | 1.074606 | 7.165 | 3.066 |  | 0.010404588 |
| Roi 3 | 60.89817 | -27.2023 | 1.0053 | 0.002695 | 0.994656 | 39.83669 | 0.80624 | 2.033 | 0.011419 | 60.16331 | 2.21764 | 1.510249 | 7.099 | 4.071 |  | 0.01064382 |
| Roi 4 | 61.00582 | -27.0024 | 1.022 | 0.00356 | 1.011533 | 41.3776 | 0.72462 | 1.968 | 0.01226 | 58.6224 | 1.78 | 1.416767 | 4.384 | 2.659 |  | 0.0104668 |
| Roi 5 | 61.11859 | -26.9973 | 1.027 | 0.00376 | 1.016586 | 49.469 | 1.023 | 1.975 | 0.0191 | 50.53065 | 2.55 | 1.021461 | 6.159 | 2.613 |  | 0.0104138 |
| Roi 6 | 61.9285 | -26.8435 | 1.037 | 0.0033 | 1.020335 | 58.891 | 0.72815 | 2.113 | 0.01513 | 41.109 | 1.295 | 0.698052 | 5.862 | 2.423 |  | 0.016664571 |
| P4 |  |  | Band-I center (µm) | Stdev(BC-I) | Band-I center (µm) corrected | B-I Area | Stdev(BA-I) | Band-II center (µm) | Stdev(BC-II) | B-II Area | Stdev(BA-II) | BAR | B-I Strength | B-II Strength | Displacement | |
| Roi 1 | 62.41677 | -25.0363 | 1.01 | 0.00446 | 0.999406 | 36.9087 | 0.598 | 2.078 | 0.011 | 63.09153 | 1.2278 | 1.567785 | 2.94 | 2.55 |  | 0.010594 |
| P5 |  |  | Band-I center (µm) | Stdev(BC-I) | Band-I center (µm) corrected | B-I Area | Stdev(BA-I) | Band-II center (µm) | Stdev(BC-II) | B-II Area | Stdev(BA-II) | BAR | B-I Strength | B-II Strength | Displacement | |
| Roi 1 | 58.72465 | -24.6697 | 0.973 | 0.00292 | 0.962014 | 35.433 | 0.39565 | 2.08187 | 0.014629 | 64.56636 | 1.05398 | 1.82221 | 4.015 | 2.745 |  | 0.0109862 |

**Table S2: Mineral percentage of locations from the mare units along with the elemental oxides measurements from Kaguya and Diviner datasets.**

|  |  |  |  |  | Total |  | Kaguya |  |  |  |  |  |  | Diviner |
| --- | --- | --- | --- | --- | --- | --- | --- | --- | --- | --- | --- | --- | --- | --- |
| P1 | Plagioclase | Olivine | Opx | Cpx | Pyroxene |  | CaO | FeO | TiO2 | Al2O3 | MgO | Mg |  | SiO2 |
| Roi 1 | 66.313 | 0.72097 | 7.888 | 25.078 | 32.966 |  | 12.8888 | 9.24956 | 2.12454 | 20.4297 | 9.11 | 63.9293 |  | 48.27 |
| Roi 2 | 57.263 | 2.24698 | 14.215 | 26.276 | 40.491 |  | 11.9193 | 10.5308 | 0.711531 | 17.5489 | 10.04 | 63.1744 |  | 48.27 |
| Roi 3 | 62.058 | 1.964 | 13.26 | 22.718 | 35.978 |  | 12.6794 | 9.61315 | 2.28159 | 19.1259 | 9.15 | 63.1315 |  | 46.82 |
| Roi 4 | 58.692 | 1.68182 | 13.867 | 25.77 | 39.637 |  | 11.7892 | 10.5866 | 3.26341 | 17.4065 | 10.78 | 64.7076 |  | 46.59 |
| Roi 5 | 49.083 | 9.271 | 16.108 | 25.538 | 41.646 |  | 11.5832 | 11.1994 | 0.001 | 17.5935 | 10.42 | 62.6165 |  | 43.72 |
| Roi 6 | 61.171 | 1.997 | 16.121 | 20.711 | 36.832 |  | 11.996 | 10.407 | 1.68015 | 17.0206 | 10.04 | 63.4626 |  | 41.8 |
| Roi 7 | 64.188 | 2.082 | 12.091 | 21.639 | 33.73 |  | 12.3941 | 10.2363 | 2.10258 | 18.8371 | 9.79 | 63.2584 |  | 42.29 |
| Roi 8 | 54.987 | 8.97109 | 15.92 | 20.121 | 36.041 |  | 12.0724 | 10.1918 | 0.835292 | 16.8717 | 9.98 | 63.8073 |  | 46.16 |
| Roi 9 | 58.567 | 3.059 | 15.341 | 23.033 | 38.374 |  | 12.1167 | 10.9568 | 1.65321 | 18.1527 | 10.62 | 63.2937 |  | 44.07 |
| Roi 10 | 62.267 | 2.75497 | 13.587 | 21.392 | 34.979 |  | 12.0861 | 10.4781 | 0.948344 | 18.3731 | 10.04 | 63.3093 |  | 40.33 |
| Roi 11 | 60.804 | 2.79 | 10.425 | 25.981 | 36.406 |  | 11.5951 | 10.7146 | 1.92806 | 18.1582 | 10.11 | 63.8339 |  | 39.96 |
| Roi 12 | 53.35 | 10.424 | 23.131 | 13.095 | 36.226 |  | 11.9633 | 11.1812 | 1.6838 | 16.6567 | 10.17 | 62.0884 |  | 46.67 |
| Roi 13 | 53.563 | 4.503 | 14.716 | 27.218 | 41.934 |  | 11.784 | 10.9977 | 0.377905 | 18.2556 | 10.5 | 63.2042 |  | 45.34 |
| Roi 14 | 47.121 | 7.216 | 35.121 | 10.542 | 45.663 |  | 12.0867 | 14.3082 | 1.79152 | 19.2725 | 8.9 | 52.8573 |  | 43.93 |
| Roi 15 | 50.054 | 5.474 | 23.891 | 20.581 | 44.472 |  | 11.6009 | 12.7764 | 4.00966 | 15.6221 | 9.89 | 58.2189 |  | 46.69 |
| Roi 16 | 53.85 | 13.242 | 17.468 | 15.44 | 32.908 |  | 11.8084 | 15.0674 | 3.49234 | 16.2673 | 8.94 | 51.649 |  | 44.03 |
| Roi 17 | 61.688 | 4.756 | 20.929 | 12.627 | 33.556 |  | 11.3674 | 10.7665 | 1.686 | 17.6998 | 10.17 | 62.9634 |  | 44.95 |
| Roi 18 | 43.754 | 7.61175 | 16.082 | 31.997 | 48.079 |  | 11.8499 | 12.1996 | 0.223631 | 17.5783 | 9.91 | 59.3747 |  | 43.94 |
| P2 | Plagioclase | Olivine | Opx | Cpx | Pyroxene |  | CaO | FeO | TiO2 | Al2O3 | MgO | Mg |  | SiO2 |
| Roi 1 | 57.354 | 8.31 | 11.831 | 22.505 | 34.336 |  | 12.4963 | 8.69585 | 0.160148 | 20.5904 | 10.3 | 68.0771 |  | 43.64 |
| Roi 2 | 68.55869 | 6.46606 | 15.615 | 9.36 | 24.97525 |  | 12.7297 | 9.60579 | 1.45081 | 19.9385 | 8.97 | 62.6872 |  | 45.24 |
| Roi 3 | 59.746 | 8.637 | 22.942 | 8.675 | 31.617 |  | 10.9196 | 10.7815 | 0.909778 | 18.3261 | 10.21 | 63.0366 |  | 43.85 |
| Roi 4 | 52.646 | 12.058 | 16.521 | 18.775 | 35.296 |  | 11.4337 | 8.19159 | 0.79 | 15.3881 | 9.29 | 67.1321 |  | 44.47 |
| P3 | Plagioclase | Olivine | Opx | Cpx | Pyroxene |  | CaO | FeO | TiO2 | Al2O3 | MgO | Mg |  | SiO2 |
| Roi 1 | 61.263 | 6.306 | 20.886 | 11.545 | 32.431 |  | 12.0816 | 10.7599 | 1.37555 | 17.9567 | 10.17 | 62.9763 |  | 42.56 |
| Roi 2 | 52.558 | 6.567 | 26.967 | 13.907 | 40.874 |  | 11.9074 | 10.9942 | 1.44302 | 17.2845 | 10.37 | 62.9275 |  | 44.64 |
| Roi 3 | 64.482 | 5.372 | 17.156 | 12.989 | 30.145 |  | 12.6139 | 9.62966 | 2.12428 | 18.9041 | 9.21 | 63.266 |  | 40.79 |
| Roi 4 | 65.518 | 2.706 | 18.493 | 13.283 | 31.776 |  | 11.7527 | 10.719 | 1.86374 | 17.7932 | 10.14 | 62.9863 |  | 45.23 |
| Roi 5 | 61.281 | 3.608 | 20.07 | 15.041 | 35.111 |  | 11.8556 | 10.8228 | 1.1673 | 18.3515 | 10.13 | 62.7446 |  | 43.63 |
| Roi 6 | 58.777 | 11.799 | 14.997 | 14.427 | 29.424 |  | 12.3038 | 10.3965 | 0.05588 | 19.3665 | 10 | 63.3935 |  | 44.63 |
| P4 | Plagioclase | Olivine | Opx | Cpx | Pyroxene |  | CaO | FeO | TiO2 | Al2O3 | MgO | Mg |  | SiO2 |
| Roi 1 | 69.299 | 3.405 | 20.942 | 6.354 | 27.296 |  | 12.48 | 10.16 | 1.83 | 19.83 | 9.67 | 63.1476 |  | 45.76 |
| P5 | Plagioclase | Olivine | Opx | Cpx | Pyroxene |  | CaO | FeO | TiO2 | Al2O3 | MgO | Mg |  | SiO2 |
| Roi 1 | 69.283 | 4.07 | 17.033 | 9.614 | 26.647 |  | 12.72 | 9.65 | 1.99 | 19.73 | 8.97 | 62.595 |  | 40.77 |
|  |  |  |  |  |  |  |  |  |  |  |  |  |  |  |
|  |  |  |  |  |  |  |  |  |  |  |  |  |  |  |
|  |  |  |  |  |  |  |  |  |  |  |  |  |  |  |
|  | Max | Max | Max | Max | Max |  | Max | Max | Max | Max | Max | Max |  | Max |
| P1 | 66.313 | 13.242 | 35.121 | 31.997 | 48.079 | P1 | 12.8888 | 15.0674 | 4.00966 | 20.4297 | 10.78 | 64.7076 |  | 48.27 |
| P2 | 68.55869 | 12.058 | 22.942 | 22.505 | 35.296 | P2 | 12.7297 | 10.7815 | 1.45081 | 20.5904 | 10.3 | 68.0771 |  | 45.24 |
| P3 | 65.518 | 11.799 | 26.967 | 15.041 | 40.874 | P3 | 12.6139 | 10.9942 | 2.12428 | 19.3665 | 10.37 | 63.3935 |  | 45.23 |
|  | Min | Min | Min | Min | Min |  | Min | Min | Min | Min | Min | Min |  | Min |
| P1 | 43.754 | 0.72097 | 7.888 | 10.542 | 32.908 | P1 | 11.3674 | 9.24956 | 0.001 | 15.6221 | 8.9 | 51.649 |  | 39.96 |
| P2 | 52.646 | 6.46606 | 11.831 | 8.675 | 24.97525 | P2 | 10.9196 | 8.19159 | 0.160148 | 15.3881 | 8.97 | 62.6872 |  | 43.64 |
| P3 | 52.558 | 2.706 | 14.997 | 11.545 | 29.424 | P3 | 11.7527 | 9.62966 | 0.05588 | 17.2845 | 9.21 | 62.7446 |  | 40.79 |

References

1. Chauhan, M., Bhattacharya, S., Pathak, S. & Chauhan, P. Remote spectral-compositional analysis of basalt mineralogy at Hansteen-Billy, Moon. *Meteorit Planet Sci* **53**, 2583–2595 (2018).

2. Hapke, B. Space weathering from Mercury to the asteroid belt. *Journal of Geophysical Research: Planets* **106**, 10039–10073 (2001).

3. Pieters, C. M. *et al.* Space weathering on airless bodies: Resolving a mystery with lunar samples. *Meteoritics & Planetary Science* **35**, 1101–1107 (2000).

4. Martinot, M. *et al.* Mineralogical Diversity and Geology of Humboldt Crater Derived Using Moon Mineralogy Mapper Data. *Journal of Geophysical Research: Planets* **123**, 612–629 (2018).

5. Clark, R. N. & Roush, T. L. Reflectance spectroscopy: Quantitative analysis techniques for remote sensing applications. *J. Geophys. Res.* **89**, 6329–6340 (1984).

6. Thesniya, P. M., Rajesh, V. J. & Flahaut, J. Ages and chemistry of mare basaltic units in the Grimaldi basin on the nearside of the Moon: Implications for the volcanic history of the basin. *Meteorit Planet Sci* **55**, 2375–2403 (2020).

7. Clark, R. N., Pieters, C. M., Green, R. O., Boardman, J. W. & Petro, N. E. Thermal removal from near-infrared imaging spectroscopy data of the Moon. *J. Geophys. Res.* **116**, E00G16 (2011).

8. Kusuma, K. N., Sebastian, N. & Murty, S. V. S. Geochemical and mineralogical analysis of Gruithuisen region on Moon using M3 and DIVINER images. *Planetary and Space Science* **67**, 46–56 (2012).

9. Zhang, X. & Cloutis, E. Near-infrared Spectra of Lunar Ferrous Mineral Mixtures. *Earth and Space Science* **8**, e2020EA001153 (2021).

10. Zhang, X. *et al.* Mineralogical variation of the late stage mare basalts: MINERALS OF THE LATE STAGE MARE BASALTS. *J. Geophys. Res. Planets* **121**, 2063–2080 (2016).

11. Klima, R. L., Dyar, M. D. & Pieters, C. M. Near-infrared spectra of clinopyroxenes: Effects of calcium content and crystal structure: Near-infrared spectra of clinopyroxenes. *Meteoritics & Planetary Science* **46**, 379–395 (2011).

12. Cloutis, E. A., Gaffey, M. J., Jackowski, T. L. & Reed, K. L. Calibrations of phase abundance, composition, and particle size distribution for olivine-orthopyroxene mixtures from reflectance spectra. *J. Geophys. Res.* **91**, 11641 (1986).

13. Gaffey, M. J., Cloutis, E. A., Kelley, M. S. & Reed, K. L. *Mineralogy of Asteroids*. *Asteroids III* 183–204 (2002).

14. Lindsley, D. H. & Andersen, D. J. A two-pyroxene thermometer. *J. Geophys. Res.* **88**, A887 (1983).
